# All-Covalent Nuclease-Resistant and Hydrogel-Tethered DNA Hairpin Probes Map pN Cell Traction Forces

**DOI:** 10.1021/acsami.3c04826

**Published:** 2023-07-06

**Authors:** Sk Aysha Rashid, Yixiao Dong, Hiroaki Ogasawara, Maia Vierengel, Mark Edoho Essien, Khalid Salaita

**Affiliations:** † Department of Chemistry, Emory University, Atlanta, Georgia 30322, United States; ‡ Wallace H. Coulter Department of Biomedical Engineering, Georgia Institute of Technology and Emory University, Atlanta, Georgia 30322, United States

**Keywords:** DNA tension probes, hydrogel, mechanobiology, integrin mechanosensing, DNA hairpin sensors, mechanotransduction

## Abstract

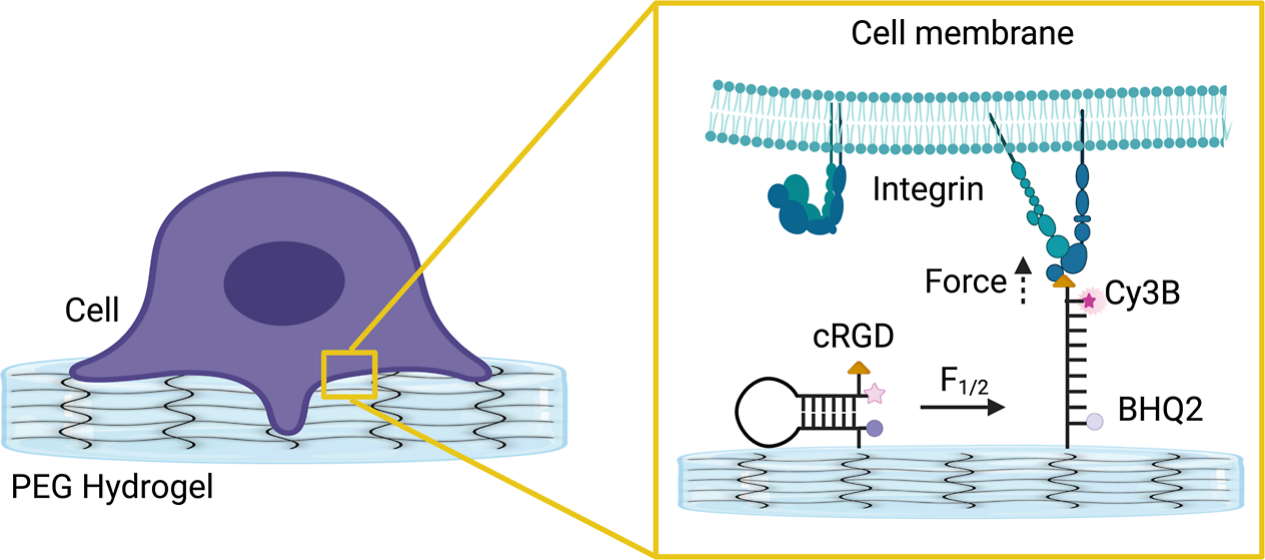

Cells sense and respond to the physical properties of their environment through receptor-mediated signaling, a process known as mechanotransduction, which can modulate critical cellular functions such as proliferation, differentiation, and survival. At the molecular level, cell adhesion receptors, such as integrins, transmit piconewton (pN)-scale forces to the extracellular matrix, and the magnitude of the force plays a critical role in cell signaling. The most sensitive approach to measuring integrin forces involves DNA hairpin-based sensors, which are used to quantify and map forces in living cells. Despite the broad use of DNA hairpin sensors to study a variety of mechanotransduction processes, these sensors are typically anchored to rigid glass slides, which are orders of magnitude stiffer than the extracellular matrix and hence modulate native biological responses. Here, we have developed nuclease-resistant DNA hairpin probes that are all covalently tethered to PEG hydrogels to image cell traction forces on physiologically relevant substrate stiffness. Using HeLa cells as a model cell line, we show that the molecular forces transmitted by integrins are highly sensitive to the bulk modulus of the substrate, and cells cultured on the 6 and 13 kPa gels produced a greater number of hairpin unfolding events compared to the 2 kPa substrates. Tension signals are spatially colocalized with pY118-paxillin, confirming focal adhesion-mediated probe opening. Additionally, we found that integrin forces are greater than 5.8 pN but less than 19 pN on 13 kPa gels. This work provides a general strategy to integrate molecular tension probes into hydrogels, which can better mimic in vivo mechanotransduction.

## Introduction

Mechanotransduction is the process by which cells sense and respond to the mechanical properties of their microenvironment. This signal transduction controls gene expression and protein activation, resulting in the regulation of various cellular processes such as cell migration, proliferation, tissue development and repair, wound healing, and maintenance of the normal tissue architecture.^1,2^ Disruption of mechanotransduction can result in various physiological imbalances and diseases, including cardiovascular diseases and cancer.^3−5^ Thus, measuring the mechanical forces generated by cells under different conditions can contribute to elucidating disease cascades and developing new therapeutic strategies.^6,7^

Interestingly, the mechanical stiffness of the extracellular matrix (ECM) plays a vital role in determining the cell fate.^8−10^ For example, increased ECM stiffness can promote tumor progression and inhibit wound healing, while decreased ECM stiffness can promote stem cell differentiation and improve the heart function after injury.^11−13^ Past studies have shown how global or local substrate stiffness can influence biological responses in a variety of cells.^9,14^ The stiffness of polymeric substrates can be tuned by changing the crosslinking density, using different hydrogel synthesis methods, and also by directly modulating the stiffness using external stimuli.^15−17^ These studies have shown that stem, neural, and muscle cells tend to have a “sweet spot” for stiffness, where cells behave poorly on too hard or soft substrates but behave optimally on ∼5–10 kPa substrates.^9,13,18−20^ On the contrary, cells usually display non-physiological stress fiber formation when cultured on a stiff substrate such as glass (<100 kPa), which is usually a sign of fibrosis.^14,21^ Past studies also revealed that endothelial cell traction forces are proportional to their substrate stiffness, with greater substrate stiffness leading to greater magnitudes of traction forces.^22^

The most widely used technique to measure cell-generated forces is traction force microscopy (TFM).^23,24^ This technique uses a soft polymer substrate doped with fluorescent beads to visualize the deformation of the gel and infer applied forces exerted by cells. One shortcoming of conventional TFM is the limited spatial and force resolution, which is generally limited to microscale and nN range force, respectively. The force magnitude resolution of TFM is 3 orders of magnitude greater than the forces generated by individual receptors which are at the pN scale.^25^ The micro-post array is another alternative technique that uses arrays of micrometer-scale pillars to measure forces generated by cells.^26,27^ However, like TFM, micro-post arrays display limited spatial resolution and require microfabrication techniques which are cumbersome.

To address the limitations of TFM and micro-post arrays, our group and others developed molecular tension probes.^28−30^ These probes are comprised of a flexible linker such as PEG,^31,32^ nucleic acids,^33^ polypeptides,^34,35^ and proteins^36^ and flanked by a fluorophore quencher pair to record mechanical extension. The probes are tethered to a surface and present ligands that can engage cell surface proteins. Thus, molecular tension probes enable one to use a conventional fluorescence microscope to measure receptor forces with pN force resolution and with optical spatial resolution. Tension sensors integrating folded DNA hairpins (HPs) are the most sensitive class of probes reported thus far and are fairly facile to generate and use.^28^ When the HP is exposed to tension, the stem-loop domain unfolds, separating a fluorophore and quencher and leading to an increase in fluorescence. These probes have been used in applications such as monitoring the mechanical properties of living cells and the study of immune cell mechanics.^33,37^ However, most past examples of DNA HPs were tethered onto a glass surface with the exception of the work by You et al., where they anchor DNA HPs onto cell membranes.^38^ Given that the mechanical properties of ECM tune cell traction force,^39^ it is desirable to investigate how molecular traction force responds to the stiffness of the substrate.

Typically, hydrogels or other polymers such as polydimethylsiloxane (PDMS) are used to investigate cell biology on compliant substrates. Several past studies by Ha and colleagues reported DNA probes tethered to such polymeric substrates.^40−42^ Typically, these studies utilized a DNA duplex probe called the tension gauge tether (TGT).^43^ However, these are irreversible and cannot capture the dynamic ECM–receptor interactions. Additionally, the studies primarily utilized biotin-streptavidin as the gel tethering group, which has an appreciable dissociation rate (2.6 × 10^–5^ s^–1^) at physiological temperatures and is prone to degradation due to proteases released by cells and present in the cell culture medium.^44,45^

To overcome these past barriers in integrin force measurement on soft substrates, we designed a hydrogel-tethered HP tension probe. Although our strategy is modular and can be adapted to work with virtually any hydrogel polymer, for our proof-of-concept, we decided to use PEG-based hydrogels because of their biocompatibility and minimal cytotoxicity of such gels.^46−48^ First, we tested various lengths of PEG precursors to prepare different moduli of hydrogels with values that are physiologically relevant. Next, we designed nuclease-resistant tension probes consisting of single-stranded phosphorothioates (PS)-modified DNA HPs. Additionally, we covalently anchored the PS-HP to the hydrogels. Because of the single-stranded structure and covalent linkage of the DNA HP probe, we observed minimal probe degradation and dissociation induced by cell-secreted nuclease and cellular traction force. We found that the number of integrin-ligand mechanical events with *F* > 5.8 pN was correlated with the rigidity of the substrate. The tension signal colocalized with pY118 paxillin, confirming that integrin forces were mediated by focal adhesions. Finally, we tested different PS DNA HP constructs (22 and 100% GC content) and found that HeLa cells cultured on 13 kPa hydrogels generated a traction force that opened the 22% GC PS-HP probe (*F* > 5.8 pN), but these forces were insufficient to open the 100% GC PS-HP, which suggests that *F* < 19 pN. This is in contrast to DNA HP tethered to glass, where *F* > 19 pN. These results confirm that the magnitudes of molecular cell traction forces are highly sensitive to the rigidity of their ECM.

## Results and Discussion

### Preparation and Characterization of the Hydrogel Scaffold

**Figure 1:**
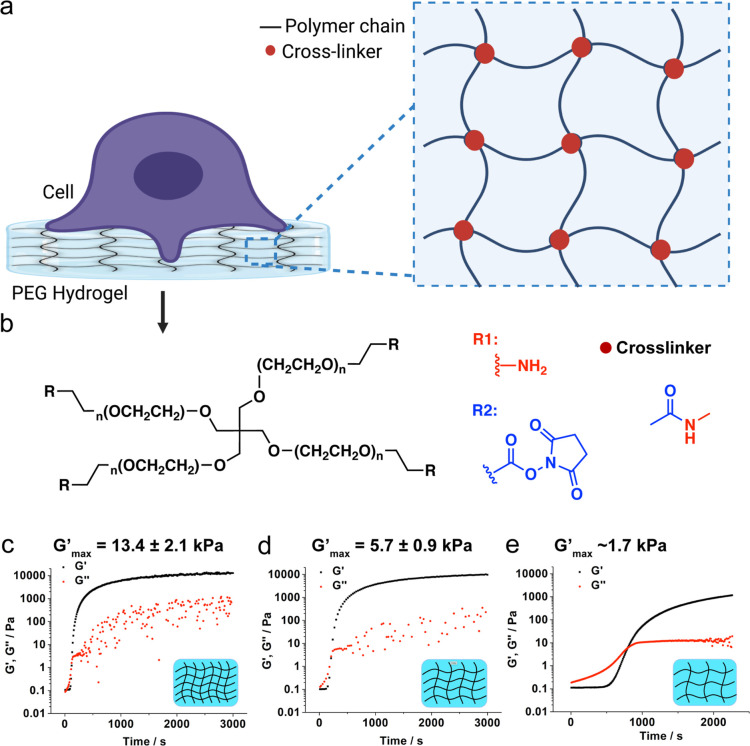
Preparation and characterization of PEG hydrogels. (a) Schematic design of working principle of hydrogel-tethered DNA hairpin probe. (b) Molecular structure of the hydrogel precursor molecules (tetra PEG-NH_2_ and tetra PEG-NHS). The PEG polymers used in our work included *n* = 28, 57, and 114 ethylene glycol monomer units, corresponding to the 5, 10, and 20 kDa polymers, respectively. (c–e) Time-dependent rheology plots of PEG hydrogels synthesized from 5 kDa PEG (c), 10 kDa PEG (d), and 20 kDa PEG (e) precursors. The maximum elastic moduli (*G*′_max_) were estimated when *G*′ reached a plateau (50 min for 5 and 10 kDa, and 90 min for the 20 kDa precursor). Note that the *G*′_max_ values were obtained from triplicate measurements for the 5 and 10 kDa polymers, whereas the *G*′_max_ for the 20 kDa polymer was a single measurement, and this was due to the slow polymerization kinetics.

To develop a generalized tool for cellular force measurement, we synthesized PEG hydrogels with a controlled modulus that mimics the softer mechanical properties of biological tissues (Figure 1a–e).

We used PEG precursors with different molecular weights to tune hydrogel stiffness. Theoretically, with the same polymer weight content (∼10 wt %), the hydrogel synthesized from lower molecular weight precursors will display a greater elastic modulus than those synthesized from greater molecular weight precursors. Briefly, three different hydrogels were synthesized with precursor molecules with 5, 10, and 20 kDa molecular weights. We chose the NHS-amine reaction to construct the PEG hydrogel as it is spontaneous, does not require radical initiators, and shows appropriate reaction kinetics to mount the hydrogel on the glass surface. After mixing the precursor molecules, 4-arm-PEG-NH_2_ (5.5% w/v) and 4-arm-PEG-NHS (5% w/v) in potassium phosphate solution and at pH 6.4, the mixture was quickly mounted on the rheometer, where we performed time-dependent rheology measurements to monitor gelation kinetics and final properties of the hydrogels (Figure 1c–e). After allowing the two monomers to form a gel, we found that the elastic modulus *G*′_max_ of the 5, 10, and 20 kDa precursors reached a plateau, which was 13.4 ± 2.1, 5.7 ± 0.9, and 1.7 kPa, respectively. These moduli are on the softer range of physiologically relevant moduli and by happenstance are mechanically similar to tissues such as the spleen, soft palate, and brain, respectively.^49^ Note that the molecular weight of the precursor also affects the gelation kinetics, and the lower molecular weight precursors tend to gelate more rapidly than the greater molecular weight precursors (Figure 1c–e). The frequency scan in rheology indicated classic hydrogel behavior as *G*′ was significantly larger than *G*″ at all frequencies tested (Figure S1). Note that we included a slight excess of the amine-containing PEG monomer precursor to provide unreacted amine groups on the surface of the gel to accommodate covalent tethering of the DNA HP probes.

It is also important to note that we chose covalently crosslinked PEG hydrogel for our experiment to minimize the viscoelastic effect of the hydrogels. This effect is the result of irreversible shifting/migration of polymer molecular segments during constant loading of mechanical forces and could dampen the tension signal while we image the cells.^50^ In principle, the magnitude of viscoelastic effects is determined by the number of molecular segments that take part in irreversible migration. For non-covalently crosslinked hydrogels, such as agarose/gelatin/DNA, the viscoelasticity is prominent due to the weak crosslinking forces among molecular segments. External forces easily break the crosslinking sites of the hydrogel, such that the deformation cannot be restored. In contrast, for covalently crosslinked hydrogels with high crosslinking density, the irreversible migration of polymer molecular segments is highly restricted, and thus the viscoelastic behavior is typically minor in such polymers used in our study.^10^

### Design and Synthesis of PS-Modified DNA Hairpin Probes

**Figure 2:**
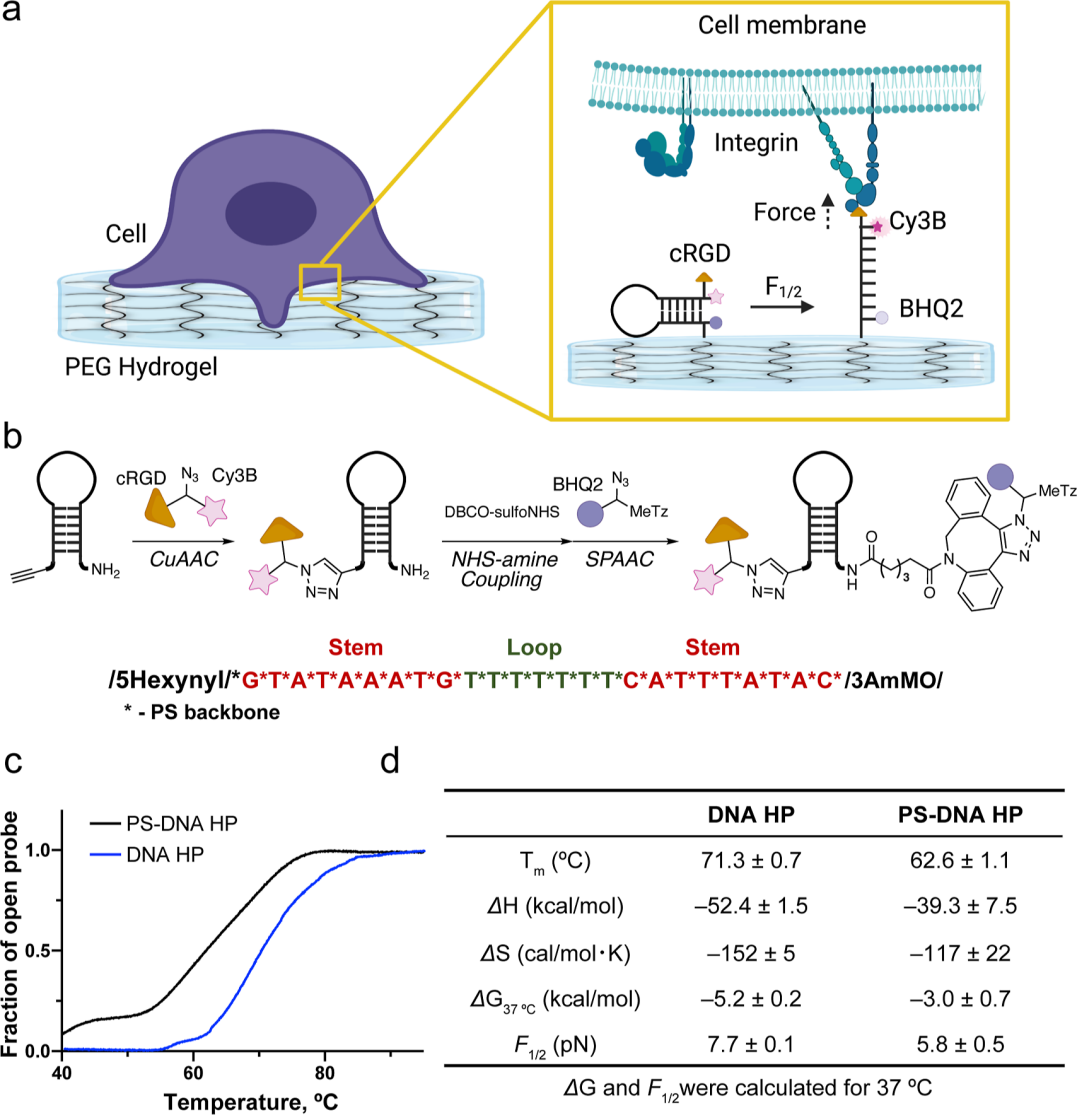
Design, synthesis, and characterization of PS-modified HP probes. (a) Schematic of a hydrogel-tethered DNA hairpin probe and its response to integrin forces. (b) Synthetic scheme for PS-modified DNA HP probes. The red color indicates the self-complementary stem, while the green color indicates the polyT loop. All nucleobases were linked by the PS backbone. (c) Fluorescence thermal melting curves of DNA HP (22% GC, blue) and PS-modified DNA HP (22% GC, black) in 1× PBS. (d) Table of the calculated *T*_m_, Δ*G*, and *F*_1/2_ for DNA HP and PS DNA HP (22% GC) at 37 °C in PBS buffer. The error values represent the standard deviation from three independent melts.

The starting material to generate the PS DNA HP probes as single-stranded nucleic acid custom synthesized with a 5′-terminal alkyne and 3′-amine modification, along with replacing the phosphodiester backbone with PS (Figure 2). The oligonucleotides were then chemically modified by a sequence of three reactions: (1) copper-catalyzed azide–alkyne cyclization (CuAAC), (2) NHS-amine coupling, and (3) strain-promoted azide–alkyne cycloaddition (SPAAC) (Figure 2b). Specifically, the 5′ alkyne terminus was coupled using CuAAC to a trifunctionalized peptide containing the cyclic(Arg-Gly-Asp-d-Phe-Lys) (cRGD) peptide, Cy3B dye along with an N_3_ group to introduce a cell-adhesive peptide and fluorophore to the 5′ end of the HP (Figure S2). A DBCO group was installed on the 3′ amine terminus, and this allowed for using a SPAAC reaction to couple the trifunctional BHQ2/tetrazine (Tz)-N_3_ to the oligonucleotide terminus (Figure 2b). The PS DNA HP probes were purified by reversed-phase HPLC and characterized by ESI-MS (Figure S3). The Gibbs free energies of hairpin unfolding for the PS-modified HP and a conventional unmodified HP were evaluated by van’t Hoff analysis using temperature-dependent fluorescence measurements in PBS (Figure 2c,d). As past studies have suggested, the PS-modified hairpin was less stable than that of conventional phosphodiester-linked DNA (Figure 2c,d).^51^ This is because PS-modifications introduce a racemic mixture of chiral centers on the DNA backbone, and hence the PS-modified DNA contains many diastereomers that weaken the duplex stability and broaden the melting transition under ensemble macroscopic analysis. Based on the thermodynamics of melting, the force at which 50% of hairpins unfold, *F*_1/2_, was calculated to be 7.7 ± 0.1 and 5.8 ± 0.5 pN at 37 °C for conventional DNA and PS-modified DNA hairpins, respectively. Note that the *F*_1/2_ calculation is based on the work of Woodside et al. 2006 and was inferred from the Gibbs free energy of unfolding and the free energy required to stretch single-strand DNA based on the worm-like chain model (Figure 2d, Supporting Information method).^33,52−54^

### Hydrogel-Tethered DNA HP Preparation and Optimization

Prior work by Ha,^41^ Leckband,^55^ Wang,^56^ and colleagues reported the use of irreversible DNA duplex-based probes that were tethered to hydrogels using either biotin-streptavidin or acrylate chemistry. We initially used this biotin-streptavidin strategy to anchor the DNA probes to the PEG hydrogel. However, in our hands, the probe density decreased dramatically (>75% loss) over 48 h (Figure S4), and seeded cells were poorly attached and spread.^45^ The lack of cell adhesion and tension signal is likely due to the *k*_off_ rate of biotin-streptavidin at 37 °C, which is 2.6 × 10^–5^ s^–1^.^44,57^ This led us to develop a covalent anchoring strategy using conventional TGT probes onto hydrogel; however, these probes showed significant nuclease-mediated probe degradation over 6 h of cell seeding (Figure S5). Thus, we decided to employ a single-stranded DNA with PS modification rather than our original three-strand design, as single-stranded PS DNA probes demonstrate high stability against traction force and nuclease activity.

**Figure 3:**
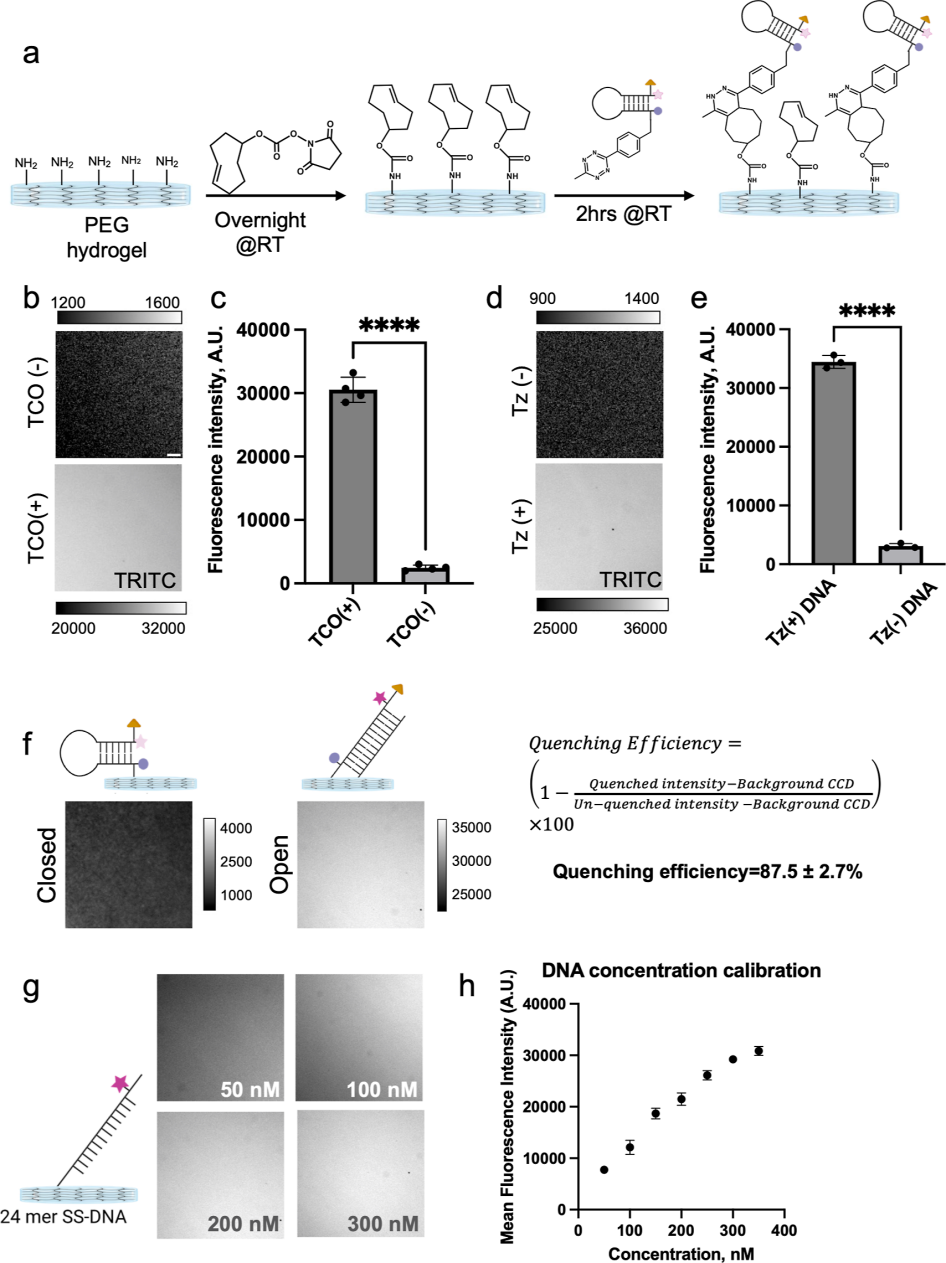
DNA hairpin surface immobilization using TCO-Tz coupling. (a) Schematic showing TCO modification of hydrogel followed by DNA HP conjugation. (b,c) Representative fluorescence images and quantification of TCO-modified hydrogels and control hydrogels following PS-DNA conjugation. (d,e) Representative fluorescence images and quantification of TCO-modified hydrogels following PS-DNA-Tz conjugation and control PS-DNA conjugation. (f) Representative fluorescence images used to determine HP quenching efficiency. As shown schematically, the fully open HP was obtained by hybridization with complement prior to surface tethering. (g,h) DNA concentration-dependent fluorescence images along with quantification show dose-dependent increase in surface density. ****, ***, **, * and ns indicate *p* < 0.0001, *p* < 0.001, *p* < 0.01, *p* < 0.05, and not significant, respectively, as determined from one-way ANOVA. Error bars show the standard deviation for *N* > 3, three different sets of surface preparations. Each intensity value was averaged from at least 10 different regions of interest. Scale bar = 10 μm.

We prepared 13 kPa PEG hydrogels with/without TCO to validate that HP binding was specific and mediated by covalent coupling (Figure 3a). The fluorescence images showed that only a subset of the probes (<5%) are non-specifically bound to the hydrogel (Figure 3b,c). We also prepared oligos without Tz modification and observed minimum nonspecific binding (<5%), further confirming specific coupling through TCO-tetrazine chemistry (Figure 3d,e). Next, we measured the quenching efficiency of the closed hairpin probes. For this experiment, we hybridized the quenched probe with a complementary oligonucleotide before reacting with the TCO-modified PEG hydrogel. Following the quenching efficiency (QE) calculation, we determined the QE to be ∼87.5%, which is slightly less efficient than our previous reports (Figure 3f).^33,58^ This lower quenching efficiency can be derived from the weaker stability of PS DNA HP at 37 °C, resulting in a slightly greater probability of probe breathing (Figure 2c,d). Finally, we titrated a range of DNA concentrations and incubation times to maximize probe density using the least amount of DNA HP reagent. We found that incubating the gels using 300 nM DNA with an incubation time of 2 h (@RT) offered the greatest fluorescence intensity in our hands for preparing hydrogel-tethered DNA HP probes (Figure 3g,h). It is important to note that the image resolution of the hydrogel tension sensors may be lower than standard tension maps on glass surfaces. This is because imaging through 100–150 μm hydrogels requires a longer working distance and lower numerical aperture (N.A.) objective lens. The lower N.A. leads to worse resolution and weaker light collecting efficiency.

### Integrins on HeLa Cells Generate *F* > 5.8 pN on PEG Hydrogels with Total Traction Forces Proportional to Substrate Stiffness

**Figure 4:**
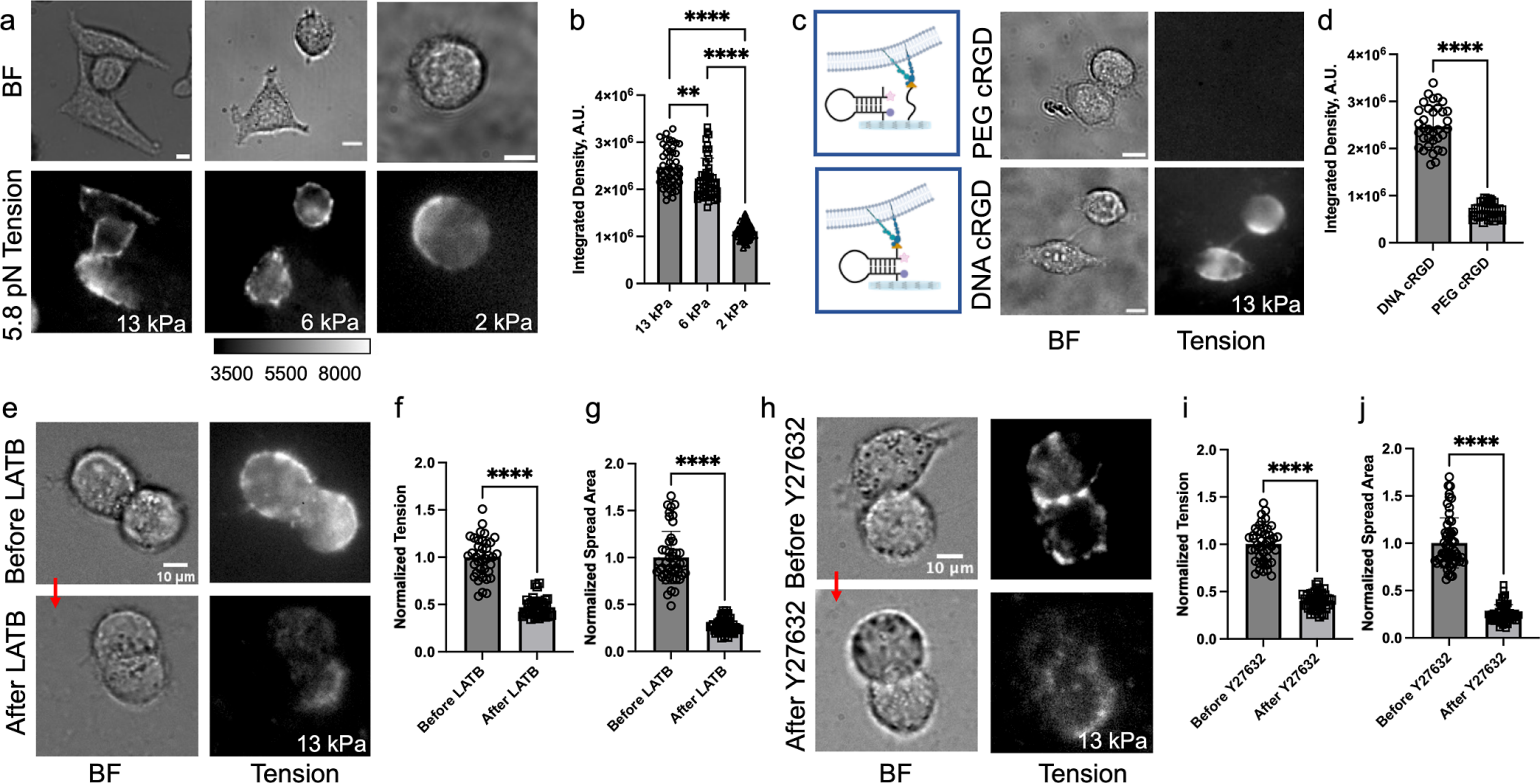
DNA HP probes map integrin tension and demonstrate enhanced traction forces in response to the Young’s modulus of the hydrogel. (a) Brightfield (BF) and fluorescence tension maps of HeLa cells cultured on 13, 6, and 2 kPa substrates coated with PS DNA HP probe (22% GC content) for ∼5–6 h. (b) Plot quantifying the total tension signal of individual HeLa cells on the different substrates. (c) Schematic illustration of probe surface and control hydrogel (left) along with BF and fluorescence tension images of HeLa cells cultured on control hydrogel coated with PEG-cRGD and cRGD-lacking PS DNA HP (top) and hydrogel coated with PS DNA HP probe (bottom). (d) Plot quantifying the total tension signal from single cells from the experiments shown in (c). (e–g) BF and tension images of HeLa cells before and after treatment with LatB (20 μM) along with bar graph quantifying single cell spread area and tension signal. Note that the plots were normalized to the before LatB treatment group. (h–j) BF and tension images of HeLa cells before and after treatment with Y27632 (25 μM) drug and bar graph quantifying single cell spread area and tension signal. Note that the plot was normalized to the non-treated cell group. ****, ***, **, *, and ns indicate *p* < 0.0001, *p* < 0.001, *p* < 0.01, *p* < 0.05, and not significant respectively, as determined from one-way ANOVA. Error bars show the standard deviation for *N* > 3, where each experiment was averaged from three or more different cell passages with three different sets of surface preparations. For each replicate *N*, we measured the signal from at least 15 cells. Scale bar = 10 μm.

In the following studies, we used HeLa cells as a model system to validate and test the hydrogel tension sensors. Due to their high nuclease and protease activity and elevated mechanical activity, HeLa cells represent a fairly challenging cell model for measuring cellular tension using conventional (unmodified) DNA probes for extended durations (Figure S6). Especially when the original three-stranded DNA HP probe was used, we observed internalized probe signal due to probe degradation or disassembly (Figure S6b,c).^33^ As described above, we prepared PS-modified hairpin-coated PEG hydrogels and incubated HeLa cells on these surfaces at 37 °C, 5% CO_2_. The cell attachment and spreading were confirmed by brightfield imaging every 60 min after the initial 3 h of incubation. Unlike cells cultured on RGD-DNA modified glass surfaces, which require 15–20 min to show cell attachment and spreading, cells cultured on the 13 kPa hydrogels required 3–6 h to spread and exert sufficient traction force to open the PS DNA HP probes (Figure 4a). After 5–6 h of cell culture, HeLa cells on the 13 kPa hydrogels showed significant fluorescence intensity, which was primarily localized in the peripheral region of each cell, which was defined by the bright field imaging. Generally, the intensity of the peripheral regions of each cell had peak values that were 2-fold greater than the background. In addition, we conducted immunostaining of phosphorylated paxillin (pY118-paxillin), which is a marker of early focal adhesion. We observed colocalization of the tension signal and pY118-paxillin, indicating that focal adhesions mediated the opening of the PS DNA HP probes (Figure S7). To evaluate the impact of substrate stiffness on the cell traction force, we quantified the fluorescence tension signal and spreading area of the HeLa cells cultured on the three hydrogels with stiffness of 2, 6, and 13 kPa. The results showed a correlation between the spreading area, tension signal, and gel stiffness. Indeed, we observed the lowest tension signal on the 2 kPa hydrogel and the greatest tension signal on the 13 kPa hydrogel (Figures 4a,b and S8). Note that past literature showed that cells cultured on stiffer substrates form a larger number of integrin clusters than the cells cultured on softer substrates (0.01–100 kPa), and it affects cell mechanosensing.^59^ However, our work focused on a narrow range of substrate stiffness (2–13 kPa), and hence we expected that the difference in integrin force is not due to the change in integrin clustering but rather driven by the difference in substrate stiffness. To rule out differences in DNA/ligand density, we prepared stiffness-varying hydrogels similarly (with the same DNA concentration and incubation time) and confirmed the DNA density by measuring background intensity before seeding cells (Figure S9). We also observed elongated F-actin expression when HeLa cells were cultured on the 13 and 6 kPa substrates compared to the 2 kPa substrate (Figure S10). This result suggests that F-actin alignment is substrate stiffness-dependent and contributes to the higher tension signal on stiffer substrates. To validate that the fluorescence signal is driven by specific forces between integrins and the probe-conjugated cRGD on the hydrogel, we created a control hydrogel substrate modified with the PS-DNA HP probes lacking cRGD, but to maintain cell adhesion, we directly grafted the cRGD peptide to the hydrogel (Figure 4c). Cells spread similarly on such control hydrogels, but the fluorescence intensity was diminished (10-fold decrease), and this value was comparable to the background intensity (Figures 4d and S8), thus confirming that the fluorescence signal is primarily mediated by specific integrin-cRGD interactions. Finally, to demonstrate the reversibility of the tension signal, we treated the cells with the F-actin inhibitor (Latrunculin B) and the Rho kinase inhibitor (Y27632) (Figure 4e–j). The treated cells showed ∼50% less spread area and ∼80% less tension signal compared to untreated cells. This confirms that the reported tension is reversible and mediated by F-actin and myosin activity in alignment with prior literature.^60−62^ Note that the rate of hairpin closing (*k*_close_) is exponentially dependent on the magnitude of applied force, and as the external force drops a few pN below *F*_1/2_, refolding rates will approach the ms time scale.^63^ Thus, the tension signal decreased immediately upon inhibitor treatment.

### HeLa Cell Integrins Generate *F* < 19 pN on 13 kPa Hydrogels

Previous work from our laboratory has shown that increasing the GC content in the stem region of DNA HPs can result in greater *F*_1/2_.^33^ Thus, we synthesized 100% GC stem-content PS DNA HP probes to evaluate the magnitude of integrin-mediated traction force when cells are cultured on soft substrates. In contrast to PS DNA HP with 22% GC showing a fluorescence melting curve (*T*_m_ = 62.6 ± 1.1 °C, Figure 2c,d), PS DNA HP with 100% GC did not show the full melting curve as higher GC content leads to significant thermodynamic stability. Because our observation and previous work suggest that PS modification decreases the duplex stability, we expected the *F*_1/2_ of the PS DNA HP probe with 100% GC to be smaller than that of the unmodified DNA HP probe (*F*_1/2_ = 19 pN), but the precise value is not clear as we were unable to obtain the full melting transition of this construct.

**Figure 5:**
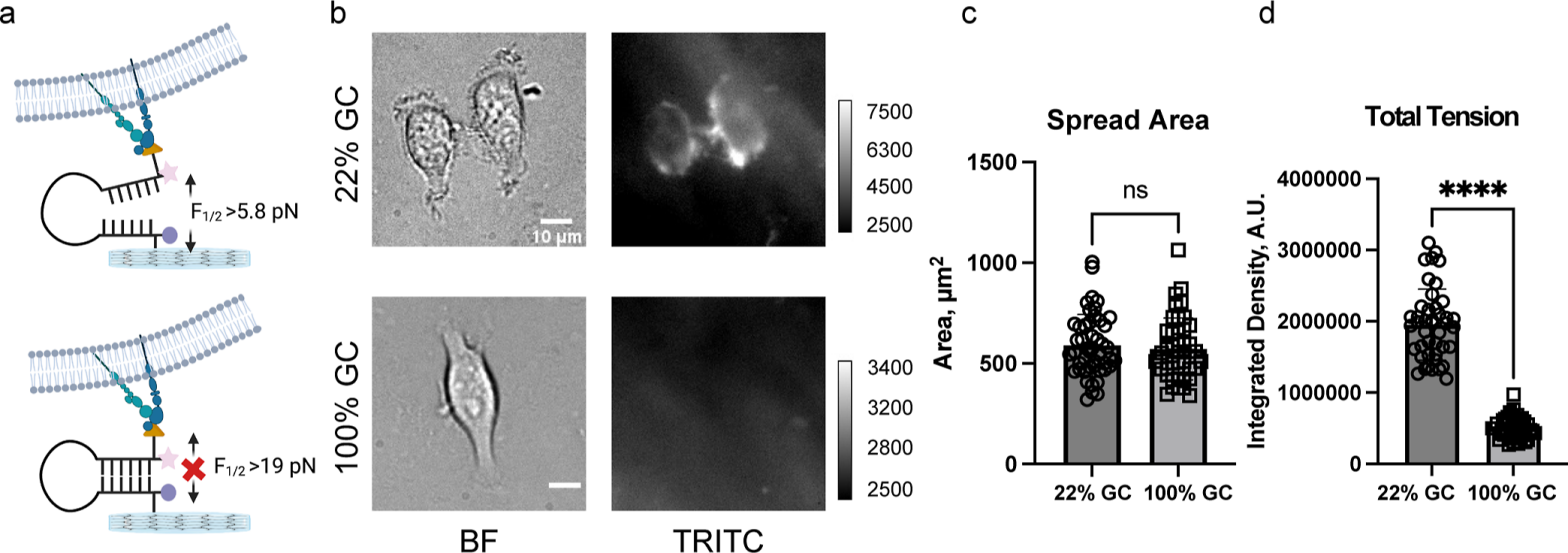
Forces generated by integrins in HeLa cells are 5.8 pN < *F*_1/2_ < 19 pN. (a) Schematic representation showing force-mediated unfolding of 22% GC PS DNA HP probes but not the 100% GC probes. (b) Brightfield and fluorescence tension (Cy3B) images of Hela cells on 5.8 and 19 pN 13 kPa hydrogel surfaces. Images were taken after ∼5–6 h of incubation. (c) Bar graph showing single cell spread area on 22 and 100% GC hairpin surfaces. (d) Bar graphs plotting the integrated fluorescence tension signal of single HeLa cells cultured on hydrogel-tethered 22 and 100% GC content PS DNA HP probes. ****, ***, **, *, and ns indicate *p* < 0.0001, *p* < 0.001, *p* < 0.01, *p* < 0.05, and not significant, respectively, as determined from one-way ANOVA. Error bars show the standard deviation for *N* > 3, where each experiment was averaged from three or more different cell passages with three different sets of surface preparations. For Each replicate *N*, at least 15 cells were quantified. Scale bar = 10 μm.

When HeLa cells were cultured on 100% GC probes, cells could not unfold the HP probes, and thus we did not observe a fluorescent tension signal. While the cell spreading areas were identical for the cells cultured on PS DNA HP probes (22 and 100% GC content), the quantified fluorescence tension signal for PS DNA HP probes (100% GC content) was much smaller than that for the PS DNA HP probes (22% GC content) (Figure 5b–d). These results suggest that the significant decrease in tension signal was not caused by poor spreading but rather this was caused by the greater magnitude of mechanical stability of the 100% GC content HP probes. Interestingly, HeLa cells cultured on a glass substrate coated with a PS DNA HP probe (100% GC) showed an observable fluorescence tension signal within 45 min of culture (Figure S11).

## Conclusions

In summary, we have developed an approach to perform molecular force measurements on soft hydrogel surfaces. The strategy involves the synthesis of all-covalent DNA that is PS-linked and nuclease resistant. The probe was coupled to the surface using the TCO-Tz click reaction, which is highly efficient and affords a mechanically robust bond resistant to proteases and nucleases. We found that these modified probes were critical in measuring hydrogel forces because of the long time scales for cell adhesion maturation on soft substrates. This strategy is modular and can be used to measure traction forces on any synthetic or natural gel, such as polyacrylamide, PDMS, collagen, and matrigel. One would need to introduce the TCO functional group onto the gel surface similarly to the NHS-TCO linker used here. We observed that integrin force is modulated by substrate stiffness and is enhanced on stiffer substrates. Molecular force measurements are consistent with previously reported TFM data measured on substrates of 1–10 kPa.^22^ Furthermore, while HeLa cells cultured on glass substrates could unfold the 100% GC content PS DNA HP, HeLa cells cultured on 13 kPa hydrogels could not unfold the probe, highlighting how the bulk substrate mechanical properties regulate molecular scale biophysical forces.

One possible limitation of applying this strategy in other 3D hydrogels that are biodegradable is the potential for proteolysis of the gel to lead to internalized probe, which would contribute to the background fluorescence signal. This process is especially problematic in cancer cell models, as such cells produce more significant amounts of proteases.^64,65^ Nonetheless, integrating nuclease-resistant all-covalent probes into hydrogels represents an important step toward measuring biophysical forces within more physiologically relevant contexts. We are now working on expanding our probe library with different GC contents and different fluorophores. Future studies will further address integrin force thresholds on the biologically relevant substrates.

## Materials and Methods

### Synthesis of PS-Modified Tension Probes

To synthesize PS-modified tension probes, we employed a three-step sequential process involving a copper-mediated azide–alkyne cyclization reaction (CuAAC), an NHS-amine coupling reaction, and a strain-promoted azide–alkyne cycloaddition reaction (SPAAC). Initially, we obtained 5′ alkyne- and 3′ amine-modified oligonucleotide with full PS-modification from Integrated DNA Technology (Coralville, IA). This oligonucleotide was then subjected to a CuAAC reaction with cRGD/Cy3B-N_3_ to introduce a cell-adhesive peptide and a fluorophore for the tension signal. Subsequently, the amine group of the PS-modified oligonucleotide was reacted with DBCO-sulfo-NHS (Click Chemistry Tools) to introduce a strained alkyne group. Finally, the strained alkyne group was reacted with BHQ2/Tz-N_3_, which acted as a quencher and surface anchoring moiety, forming the PS-modified tension probe. The detailed synthetic scheme can be found in the Supporting Information.

### Synthesis of the Hydrogel Surface

To create the PEG hydrogel surface, two precursor solutions were mixed. In the case of the hydrogel 5kDa molecular weight precursor, the first solution contained 55 mg of 4-arm-PEG-NH_2_ (*M*_w_ 5kDa; Biopharma PEG, 10,225) dissolved in 500 μL potassium phosphate buffer (pH 6.4), while the second solution contained 50 mg of the 4-arm-PEG-NHS molecule (*M*_w_ 5kDa; NOF America Corp. PTE05GS) dissolved in 500 μL potassium phosphate buffer (pH 6.4). The solutions were then cooled on ice for 15 min and mixed vigorously using a stir bar for 5 s at approximately 400 rpm. A 20 μL mixture was then placed between a parafilm strip and an APTES-treated glass surface to create a thin (80 μm) hydrogel layer, which was incubated for 1 h at room temperature. Please note that the molecular weight given for 4-arm-PEG molecules refers to the overall molecular weight, not that of one arm. For example, a 4-arm-PEG molecule with a total molecular weight of 5kDa would have an *M*_w_ 5000/4 = 1250 for each arm.

### Gel Stiffness Characterization

The rheological properties of PEG hydrogel precursor mixtures were evaluated using an AR2000ex rheometer equipped with a temperature-controlled stage. The measurements were conducted between a 25 mm parallel plate and a stainless-steel stage using 100 μL of the liquid precursor mixture. The gap size was set to 0.1 mm. Oscillation mode was employed, where the top plate oscillated back and forth with a fixed amplitude and frequency. Time-dependent rheological experiments were performed at 25 °C with a constant strain of 1% and a frequency of 1 Hz. Frequency sweep tests were performed on mixtures ranging from 0.01 to 1000 Hz at 25 °C and a fixed strain of 1%.

### Surface Preparation

The parafilm strip was then removed by using tweezers, and the gel was treated with DMSO for 5 min to wash and incubated overnight with 200 μL of 5 mg/mL TCO-NHS ester (BroadPharm, CA) in DMSO. After thoroughly washing the TCO-coated PEG surface with DMSO (3 times), EtOH (3 times), nano-pure water (3 times), and 1× PBS (3 times), 300 nM DNA was incubated on the surface for 2 h. The gel was washed thoroughly with PBS (3 times) and cell culture media (3 times) before seeding.

### Cell Imaging

The imaging of HeLa cells was performed in a 5% serum-containing DMEM at a temperature of 25 °C, utilizing a Nikon Eclipse Ti microscope controlled by the Elements software package. This advanced microscope was equipped with various components, including an Evolve electron-multiplying charge-coupled device manufactured by Photometrics, an Intensilight epifluorescence source produced by Nikon, and a CFI Apo 40× objective, also made by Nikon. The confocal measurements were conducted on a Nikon Ti Eclipse Inverted confocal microscope using a Plan Apo Lambda 40×/1.40 objective lens and Nikon Elements 4.40.00 software. In addition, the microscope was equipped with a C2 Laser launch consisting of 405 and 561 nm diode lasers.

### Cell Culture

We employed HeLa cells for our experiments. To provide optimal culture conditions, we used a culture medium with 10% fetal bovine serum (FBS) supplemented with 2.2 mM l-glutamine and 1% antibiotic. To initiate cell adhesion and to spread, we added the cells to the substrate and incubated them for 3–6 h at 37 °C with 5% serum and 1% antibiotic. During this time, the cells attached to the surface began to spread, allowing us to capture images and videos of their behavior. Our experiments required careful control of environmental conditions to ensure the viability and functionality of the cells.

### Immunostaining

The cells were fixed by adding 2–4% formaldehyde in 1× PBS for 8–10 min. After fixation, cells were permeabilized with 0.1% Triton X-100 for 3 min and then blocked with BSA for 30 min. To perform staining, cells were exposed to 1:1000 Alexa 488-Phalloidin (Actin staining, ab176753, Abcam), 1:50 Phospho-Paxillin (Tyr118) Polyclonal Antibody (PA5-17828, Thermo Fisher and Antibody 2541, Cell signaling). The cells were incubated with the primary antibodies for 1 h at room temperature or refrigerated overnight, followed by 1:1000 Alexa Fluor 647 or 488 goat anti-mouse IgG2b (γ2b) (A28175 or A28181) or goat anti-rabbit secondary antibody (A27034 or A27080) from Thermo Fisher as indicated in the experiment details. The immunostained cells were then imaged with fluorescence microscopy.

### Drug Treatment

Before seeding, the HeLa cells were subjected to various inhibitors for over 30 min to 1 h at room temperature. The cells were exposed to different treatments, including 25 μM Y27632 dihydrochloride (Y0503, MilliporeSigma) for 30 min to 1 h or 20 μM latrunculin B for 30 min to 1 h. The control group was treated with 0.2% DMSO, which was used as a solvent vehicle.

### Illustration and Statistical Analysis

All the illustrations of this manuscript were prepared by either Adobe Illustrator or https://www.biorender.com/. Statistical analyses were performed in GraphPad and ImageJ.

## Supporting Information Available

Additional data, statistical analysis, and details on experimental procedures (PDF)

## References

[ref1] Mohammed D.; Versaevel M.; Bruyère C.; Alaimo L.; Luciano M.; Vercruysse E.; Procès A.; Gabriele S. Innovative Tools for Mechanobiology: Unraveling Outside-in and inside-out Mechanotransduction. Front. Bioeng. Biotechnol. 2019, 7, 162 10.3389/fbioe.2019.00162. 31380357 PMC6646473

[ref2] Martino F.; Perestrelo A. R.; Vinarský V.; Pagliari S.; Forte G. Cellular Mechanotransduction: From Tension to Function. Front. Physiol. 2018, 9, 824 10.3389/fphys.2018.00824. 30026699 PMC6041413

[ref3] Jaalouk D. E.; Lammerding J. Mechanotransduction Gone Awry. Nat. Rev. Mol. Cell Biol. 2009, 10, 63–73. 10.1038/nrm2597. 19197333 PMC2668954

[ref4] Chin L.; Xia Y.; Discher D. E.; Janmey P. A. Mechanotransduction in Cancer. Curr. Opin. Chem. Eng. 2016, 11, 77–84. 10.1016/j.coche.2016.01.011. 28344926 PMC5362117

[ref5] Jansen K. A.; Atherton P.; Ballestrem C. Mechanotransduction at the Cell-Matrix Interface. Semin. Cell Dev. Biol. 2017, 71, 75–83. 10.1016/j.semcdb.2017.07.027. 28754442

[ref6] Dombroski J. A.; Hope J. M.; Sarna N. S.; King M. R. Channeling the Force: Piezo1 Mechanotransduction in Cancer Metastasis. Cells 2021, 10, 2815 10.3390/cells10112815. 34831037 PMC8616475

[ref7] Jiang Y.; Zhang H.; Wang J.; Liu Y.; Luo T.; Hua H. Targeting Extracellular Matrix Stiffness and Mechanotransducers to Improve Cancer Therapy. J. Hematol. Oncol. 2022, 15, 34 10.1186/s13045-022-01252-0. 35331296 PMC8943941

[ref8] Riehl B. D.; Kim E.; Bouzid T.; Lim J. Y. The Role of Microenvironmental Cues and Mechanical Loading Milieus in Breast Cancer Cell Progression and Metastasis. Front. Bioeng. Biotechnol. 2021, 8, 608526 10.3389/fbioe.2020.608526. 33585411 PMC7874074

[ref9] Ge H.; Tian M.; Pei Q.; Tan F.; Pei H. Extracellular Matrix Stiffness: New Areas Affecting Cell Metabolism. Front. Oncol. 2021, 11, 631991 10.3389/fonc.2021.631991. 33718214 PMC7943852

[ref10] Chaudhuri O.; Cooper-White J.; Janmey P. A.; Mooney D. J.; Shenoy V. B. Effects of Extracellular Matrix Viscoelasticity on Cellular Behaviour. Nature 2020, 584, 535–546. 10.1038/s41586-020-2612-2. 32848221 PMC7676152

[ref11] Wang M.; Yang Y.; Han L.; Han S.; Liu N.; Xu F.; Li F. Effect of Three-Dimensional ECM Stiffness on Cancer Cell Migration through Regulating Cell Volume Homeostasis. Biochem. Biophys. Res. Commun. 2020, 528, 459–465. 10.1016/j.bbrc.2020.05.182. 32505356

[ref12] Frangogiannis N. G. The Extracellular Matrix in Myocardial Injury, Repair, and Remodeling. J. Clin. Invest. 2017, 127, 1600–1612. 10.1172/jci87491. 28459429 PMC5409799

[ref13] Smith L. R.; Cho S.; Discher D. E. Stem Cell Differentiation Is Regulated by Extracellular Matrix Mechanics. Physiology 2018, 33, 16–25. 10.1152/physiol.00026.2017. 29212889 PMC5866410

[ref14] Yi B.; Xu Q.; Liu W. An Overview of Substrate Stiffness Guided Cellular Response and Its Applications in Tissue Regeneration. Bioact. Mater. 2022, 15, 82–102. 10.1016/j.bioactmat.2021.12.005. 35386347 PMC8940767

[ref15] Guo J.; Shu X.; Deng H.; Zhang J.; Wang Y.; Meng G.; He J.; Wu F. Stiff and Tough Hydrogels Prepared through Integration of Ionic Cross-Linking and Enzymatic Mineralization. Acta Biomater. 2022, 149, 220–232. 10.1016/j.actbio.2022.06.008. 35688402

[ref16] Luo T.; Tan B.; Zhu L.; Wang Y.; Liao J. A Review on the Design of Hydrogels with Different Stiffness and Their Effects on Tissue Repair. Front. Bioeng. Biotechnol. 2022, 10, 817391 10.3389/fbioe.2022.817391. 35145958 PMC8822157

[ref17] Ting M. S.; Travas-Sejdic J.; Malmstrom J. Modulation of Hydrogel Stiffness by External Stimuli: Soft Materials for Mechanotransduction Studies. J. Mater. Chem. B 2021, 9, 7578–7596. 10.1039/d1tb01415c. 34596202

[ref18] Boothe S. D.; Myers J. D.; Pok S.; Sun J.; Xi Y.; Nieto R. M.; Cheng J.; Jacot J. G. The Effect of Substrate Stiffness on Cardiomyocyte Action Potentials. Cell Biochem. Biophys. 2016, 74, 527–535. 10.1007/s12013-016-0758-1. 27722948 PMC5102789

[ref19] Corbin E. A.; Vite A.; Peyster E. G.; Bhoopalam M.; Brandimarto J.; Wang X.; Bennett A. I.; Clark A. T.; Cheng X.; Turner K. T.; Musunuru K.; Margulies K. B.; Margulies K. B. Tunable and Reversible Substrate Stiffness Reveals a Dynamic Mechanosensitivity of Cardiomyocytes. ACS Appl. Mater. Interfaces 2019, 11, 20603–20614. 10.1021/acsami.9b02446. 31074953 PMC13720955

[ref20] Zhang C.; Tan Y.; Feng J.; Huang C.; Liu B.; Fan Z.; Xu B.; Lu T. Exploration of the Effects of Substrate Stiffness on Biological Responses of Neural Cells and Their Mechanisms. ACS Omega 2020, 5, 31115–31125. 10.1021/acsomega.0c04279. 33324820 PMC7726759

[ref21] Liu B.; Kilpatrick J. I.; Lukasz B.; Jarvis S. P.; McDonnell F.; Wallace D. M.; Clark A. F.; O’Brien C. J. Increased Substrate Stiffness Elicits a Myofibroblastic Phenotype in Human Lamina Cribrosa Cells. Invest. Ophthalmol. Visual Sci. 2018, 59, 803–814. 10.1167/iovs.17-22400. 29392327

[ref22] Califano J. P.; Reinhart-King C. A. Substrate Stiffness and Cell Area Predict Cellular Traction Stresses in Single Cells and Cells in Contact. Cell. Mol. Bioeng. 2010, 3, 68–75. 10.1007/s12195-010-0102-6. 21116436 PMC2992361

[ref23] Hur S. S.; Jeong J. H.; Ban M. J.; Park J. H.; Yoon J. K.; Hwang Y. Traction Force Microscopy for Understanding Cellular Mechanotransduction. BMB Rep. 2020, 53, 74–81. 10.5483/bmbrep.2020.53.2.308. 31964473 PMC7061206

[ref24] Lekka M.; Gnanachandran K.; Kubiak A.; Zielinski T.; Zemla J. Traction Force Microscopy - Measuring the Forces Exerted by Cells. Micron 2021, 150, 103138 10.1016/j.micron.2021.103138. 34416532

[ref25] Plotnikov S. V.; Sabass B.; Schwarz U. S.; Waterman C. M. High-Resolution Traction Force Microscopy. Methods Cell Biol. 2014, 123, 367–394. 10.1016/B978-0-12-420138-5.00020-3. 24974038 PMC4699589

[ref26] Beussman K. M.; Rodriguez M. L.; Leonard A.; Taparia N.; Thompson C. R.; Sniadecki N. J. Micropost Arrays for Measuring Stem Cell-Derived Cardiomyocyte Contractility. Methods 2016, 94, 43–50. 10.1016/j.ymeth.2015.09.005. 26344757 PMC4761463

[ref27] Shi Y.; Sivarajan S.; Crocker J. C.; Reich D. H. Measuring Cytoskeletal Mechanical Fluctuations and Rheology with Active Micropost Arrays. Curr. Protoc. 2022, 2, e433 10.1002/cpz1.433. 35612274 PMC9321978

[ref28] Liu Y.; Galior K.; Ma V. P.; Salaita K. Molecular Tension Probes for Imaging Forces at the Cell Surface. Acc. Chem. Res. 2017, 50, 2915–2924. 10.1021/acs.accounts.7b00305. 29160067 PMC6066286

[ref29] Ma V. P.; Salaita K. DNA Nanotechnology as an Emerging Tool to Study Mechanotransduction in Living Systems. Small 2019, 15, e1900961 10.1002/smll.201900961. 31069945 PMC6663650

[ref30] Hang X.; He S.; Dong Z.; Minnick G.; Rosenbohm J.; Chen Z.; Yang R.; Chang L. Nanosensors for Single Cell Mechanical Interrogation. Biosens. Bioelectron. 2021, 179, 113086 10.1016/j.bios.2021.113086. 33636499

[ref31] Stabley D. R.; Jurchenko C.; Marshall S. S.; Salaita K. S. Visualizing Mechanical Tension across Membrane Receptors with a Fluorescent Sensor. Nat. Methods 2012, 9, 64–67. 10.1038/nmeth.1747. 22037704

[ref32] Liu Y.; Yehl K.; Narui Y.; Salaita K. Tension Sensing Nanoparticles for Mechano-Imaging at the Living/Nonliving Interface. J. Am. Chem. Soc. 2013, 135, 5320–5323. 10.1021/ja401494e. 23495954 PMC3630457

[ref33] Zhang Y.; Ge C.; Zhu C.; Salaita K. DNA-Based Digital Tension Probes Reveal Integrin Forces During Early Cell Adhesion. Nat. Commun. 2014, 5, 5167 10.1038/ncomms6167. 25342432 PMC4209443

[ref34] Morimatsu M.; Mekhdjian A. H.; Adhikari A. S.; Dunn A. R. Molecular Tension Sensors Report Forces Generated by Single Integrin Molecules in Living Cells. Nano Lett. 2013, 13, 3985–3989. 10.1021/nl4005145. 23859772 PMC3815579

[ref35] Chang A. C.; Mekhdjian A. H.; Morimatsu M.; Denisin A. K.; Pruitt B. L.; Dunn A. R. Single Molecule Force Measurements in Living Cells Reveal a Minimally Tensioned Integrin State. ACS Nano 2016, 10, 10745–10752. 10.1021/acsnano.6b03314. 27779848 PMC5886374

[ref36] Galior K.; Liu Y.; Yehl K.; Vivek S.; Salaita K. Titin-Based Nanoparticle Tension Sensors Map High-Magnitude Integrin Forces within Focal Adhesions. Nano Lett. 2016, 16, 341–348. 10.1021/acs.nanolett.5b03888. 26598972 PMC5592801

[ref37] Liu Y.; Blanchfield L.; Ma V. P.; Andargachew R.; Galior K.; Liu Z.; Evavold B.; Salaita K. DNA-Based Nanoparticle Tension Sensors Reveal That T-Cell Receptors Transmit Defined pN Forces to Their Antigens for Enhanced Fidelity. Proc. Natl. Acad. Sci. U.S.A. 2016, 113, 5610–5615. 10.1073/pnas.1600163113. 27140637 PMC4878516

[ref38] Zhao B.; O’Brien C.; Mudiyanselage A.; Li N.; Bagheri Y.; Wu R.; Sun Y.; You M. Visualizing Intercellular Tensile Forces by DNA-Based Membrane Molecular Probes. J. Am. Chem. Soc. 2017, 139, 18182–18185. 10.1021/jacs.7b11176. 29211468

[ref39] Humphrey J. D.; Dufresne E. R.; Schwartz M. A. Mechanotransduction and Extracellular Matrix Homeostasis. Nat. Rev. Mol. Cell Biol. 2014, 15, 802–812. 10.1038/nrm3896. 25355505 PMC4513363

[ref40] Wang X.; Rahil Z.; Li I. T.; Chowdhury F.; Leckband D. E.; Chemla Y. R.; Ha T. Constructing Modular and Universal Single Molecule Tension Sensor Using Protein G to Study Mechano-Sensitive Receptors. Sci. Rep. 2016, 6, 21584 10.1038/srep21584. 26875524 PMC4753514

[ref41] Chowdhury F.; Li I. T. S.; Leslie B. J.; Doganay S.; Singh R.; Wang X.; Seong J.; Lee S. H.; Park S.; Wang N.; Ha T. Single Molecular Force across Single Integrins Dictates Cell Spreading. Integr. Biol. 2015, 7, 1265–1271. 10.1039/c5ib00080g. PMC4593737 26143887

[ref42] Jo M. H.; Li J.; Jaumouille V.; Hao Y.; Coppola J.; Yan J.; Waterman C. M.; Springer T. A.; Ha T. Single-molecule characterization of subtype-specific β1 integrin mechanics. Nat. Commun. 2022, 13, 7471 10.1038/s41467-022-35173-w. 36463259 PMC9719539

[ref43] Wang X.; Ha T. Defining Single Molecular Forces Required to Activate Integrin and Notch Signaling. Science 2013, 340, 991–994. 10.1126/science.1231041. 23704575 PMC3710701

[ref44] Jurchenko C.; Chang Y.; Narui Y.; Zhang Y.; Salaita K. S. Integrin-Generated Forces Lead to Streptavidin-Biotin Unbinding in Cellular Adhesions. Biophys. J. 2014, 106, 1436–1446. 10.1016/j.bpj.2014.01.049. 24703305 PMC3976516

[ref45] Rashid S. A.; Blanchard A. T.; Combs J. D.; Fernandez N.; Dong Y.; Cho H. C.; Salaita K. DNA Tension Probes Show That Cardiomyocyte Maturation Is Sensitive to the Piconewton Traction Forces Transmitted by Integrins. ACS Nano 2022, 16, 5335–5348. 10.1021/acsnano.1c04303. 35324164 PMC11238821

[ref46] Bjugstad K. B.; Lampe K.; Kern D. S.; Mahoney M. Biocompatibility of Poly(Ethylene Glycol)-Based Hydrogels in the Brain: An Analysis of the Glial Response across Space and Time. J. Biomed. Mater. Res., Part A 2010, 95A, 79–91. 10.1002/jbm.a.32809. 20740603

[ref47] Zhu J. Bioactive Modification of Poly(Ethylene Glycol) Hydrogels for Tissue Engineering. Biomaterials 2010, 31, 4639–4656. 10.1016/j.biomaterials.2010.02.044. 20303169 PMC2907908

[ref48] Gao R.; Yu C. C.; Gao L.; Piatkevich K. D.; Neve R. L.; Munro J. B.; Upadhyayula S.; Boyden E. S. A Highly Homogeneous Polymer Composed of Tetrahedron-Like Monomers for High-Isotropy Expansion Microscopy. Nat. Nanotechnol. 2021, 16, 698–707. 10.1038/s41565-021-00875-7. 33782587 PMC8197733

[ref49] Singh G.; Chanda A. Mechanical Properties of Whole-Body Soft Human Tissues: A Review. Biomed. Mater. 2021, 16, 062004 10.1088/1748-605x/ac2b7a. 34587593

[ref50] Hajikarimi P.; Moghadas Nejad F. Chapter 3—Mechanical Models of Viscoelasticity. In Applications of Viscoelasticity; Hajikarimi P., Moghadas Nejad F., Ed.; Elsevier, 2021; pp 27–61.

[ref51] Kibler-Herzog L.; Zon G.; Uznanski B.; Whittier G.; Wilson W. D. Duplex Stabilities of Phosphorothioate, Methylphosphonate, and RNA Analogs of Two DNA 14-mers. Nucleic Acids Res. 1991, 19, 2979–2986. 10.1093/nar/19.11.2979. 1711677 PMC328260

[ref52] Du Q.; Smith C.; Shiffeldrim N.; Vologodskaia M.; Vologodskii A. Cyclization of Short DNA Fragments and Bending Fluctuations of the Double Helix. Proc. Natl. Acad. Sci. U.S.A. 2005, 102, 5397–5402. 10.1073/pnas.0500983102. 15809441 PMC556251

[ref53] Brockman J. M.; Su H.; Blanchard A. T.; Duan Y.; Meyer T.; Quach M. E.; Glazier R.; Bazrafshan A.; Bender R. L.; Kellner A. V.; Ogasawara H.; Ma R.; Schueder F.; Petrich B. G.; Jungmann R.; Li R.; Mattheyses A. L.; Ke Y.; Salaita K. Live-Cell Super-Resolved PAINT Imaging of Piconewton Cellular Traction Forces. Nat. Methods 2020, 17, 1018–1024. 10.1038/s41592-020-0929-2. 32929270 PMC7574592

[ref54] Woodside M. T.; Behnke-Parks W. M.; Larizadeh K.; Travers K.; Herschlag D.; Block S. M. Nanomechanical Measurements of the Sequence-Dependent Folding Landscapes of Single Nucleic Acid Hairpins. Proc. Natl. Acad. Sci. U.S.A. 2006, 103, 6190–6195. 10.1073/pnas.0511048103. 16606839 PMC1458853

[ref55] Rahil Z.; Pedron S.; Wang X.; Ha T.; Harley B.; Leckband D. Nanoscale Mechanics Guides Cellular Decision Making. Integr. Biol. 2016, 8, 929–935. 10.1039/c6ib00113k. PMC5021613 27477049

[ref56] Sarkar A.; LeVine D.; Zhao Y.; Mollaeian K.; Ren J.; Wang X. Tandem Tension Sensor Reveals Substrate Rigidity-Dependence of Integrin Molecular Tensions in Live Cells, 2020, bioRxiv: 2020.01.24.918946.

[ref57] Deng L.; Kitova E. N.; Klassen J. S. Dissociation Kinetics of the Streptavidin–Biotin Interaction Measured Using Direct Electrospray Ionization Mass Spectrometry Analysis. J. Am. Soc. Mass Spectrom. 2013, 24, 49–56. 10.1007/s13361-012-0533-5. 23247970

[ref58] Glazier R.; Shinde P.; Ogasawara H.; Salaita K. Spectroscopic Analysis of a Library of DNA Tension Probes for Mapping Cellular Forces at Fluid Interfaces. ACS Appl. Mater. Interfaces 2021, 13, 2145–2164. 10.1021/acsami.0c09774. 33417432

[ref59] Cheng B.; Wan W.; Huang G.; Li Y.; Genin G. M.; Mofrad M. R. K.; Lu T. J.; Xu F.; Lin M. Nanoscale Integrin Cluster Dynamics Controls Cellular Mechanosensing Via Faky397 Phosphorylation. Sci. Adv. 2020, 6, eaax1909 10.1126/sciadv.aax1909. 32181337 PMC7056303

[ref60] Doss B. L.; Pan M.; Gupta M.; Grenci G.; Mege R. M.; Lim C. T.; Sheetz M. P.; Voituriez R.; Ladoux B. Cell Response to Substrate Rigidity Is Regulated by Active and Passive Cytoskeletal Stress. Proc. Natl. Acad. Sci. U.S.A. 2020, 117, 12817–12825. 10.1073/pnas.1917555117. 32444491 PMC7293595

[ref61] Ghabache E.; Cao Y.; Miao Y.; Groisman A.; Devreotes P. N.; Rappel W. J. Coupling Traction Force Patterns and Actomyosin Wave Dynamics Reveals Mechanics of Cell Motion. Mol. Syst. Biol. 2021, 17, e10505 10.15252/msb.202110505. 34898015 PMC8666840

[ref62] Ellefsen K. L.; Holt J. R.; Chang A. C.; Nourse J. L.; Arulmoli J.; Mekhdjian A. H.; Abuwarda H.; Tombola F.; Flanagan L. A.; Dunn A. R.; Parker I.; Pathak M. M. Myosin-II Mediated Traction Forces Evoke Localized Piezo1-Dependent Ca(2+) Flickers. Commun. Biol. 2019, 2, 298 10.1038/s42003-019-0514-3. 31396578 PMC6685976

[ref63] Su H.; Brockman J. M.; Duan Y.; Sen N.; Chhabra H.; Bazrafshan A.; Blanchard A. T.; Meyer T.; Andrews B.; Doye J. P. K.; Ke Y.; Dyer R. B.; Salaita K. Massively Parallelized Molecular Force Manipulation with on-Demand Thermal and Optical Control. J. Am. Chem. Soc. 2021, 143, 19466–19473. 10.1021/jacs.1c08796. 34762807 PMC9066152

[ref64] López-Otín C.; Bond J. S. Proteases: Multifunctional Enzymes in Life and Disease. J. Biol. Chem. 2008, 283, 30433–30437. 10.1074/jbc.r800035200. 18650443 PMC2576539

[ref65] Lee M.; Fridman R.; Mobashery S. Extracellular Proteases as Targets for Treatment of Cancer Metastases. Chem. Soc. Rev. 2004, 33, 401–409. 10.1039/b209224g. 15354221

