# Supplementary material for: All-Covalent Nuclease-Resistant and Hydrogel-Tethered DNA Hairpin Probes Map pN Cell Traction Forces
Source: ACS Appl Mater Interfaces. 2023 Jul 6;15(28):33362–72. doi: 10.1021/acsami.3c04826 (PMC10360067; doi:10.1021/acsami.3c04826)
Supplement: Supplementary file 1 — am3c04826_si_001.pdf [file am3c04826_si_001.pdf]

Supporting Information

All-covalent Nuclease-resistant and Hydrogel-tethered DNA Hairpin Probes Map pN Cell Traction Forces

*Sk Aysha Rashid[1#]; Yixiao Dong[1#]; Hiroaki Ogasawara[1]; Maia Vierengel[1]; Mark Edoho Essien[1]; and Khalid Salaita[1,2]**

#These authors contributed equally.

[1]Department of Chemistry, Emory University, Atlanta, GA 30322, USA

[2]Wallace H. Coulter Department of Biomedical Engineering, Georgia Institute of Technology and Emory University, Atlanta, GA 30322, USA.

To whom correspondence may be addressed. Email: k.salaita@emory.edu

Table S1: Sequence of DNA-HP probes used in this study.

Name	Sequence (5' to 3')
PS 22% GC	/5Hexynyl/*G*T* A*T*A* A*A*T* G*T*T* T*T*T* T*T*C* A*T*T* T*A*T* A*C*/3AmMO/
PS 100% GC	/5Hexynyl/*G*C* G*C*G* C*G*C* G*C*G* C*T*T* T*T*G* C*G*C* G*C*G* C*G*C* G*C*/3AmMO/

*Phosphorothioates (PS) modification

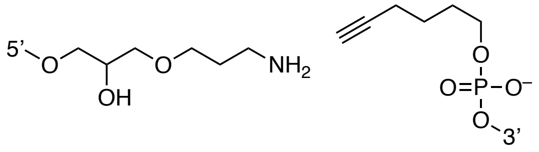

3AmO 5Hexynyl

1. **Copper-mediated azido-alkyne cyclization reaction for alkyne-modified oligonucleotides (22% GC hairpin & 100% GC hairpin)**

cRGD-Lys(N_3)-Cy3B (cRGD/Cy3B-N_3) was ligated to the corresponding 5' alkyne-modified strand (IDT) via 1,3-dipolar cycloaddition reaction. Briefly, a solution of 10 nmol of phosphorothioate-modified DNA was reacted 1 hour at 50 ºC with 20 nmol of azido reagents in the presence of sodium ascorbate (0.10 µmol), $CuSO_4$ (0.10 µmol), THPTA (0.75 µmol), and triethyl amine (4 µmol) in 45 µL (3: 2 = 18.2 MΩ MilliQ water: DMSO). The product was filtered through a microcentrifuge filter (0.22 µm). The filtrate was subjected to reverse-phase HPLC (AdvancedBio Oligonucleotides, Agilent, 4.6 × 150 mm, 0.5 mL min^{-1} flow rate; solvent A: 0.1 M triethylammonium acetate (TEAA), solvent B: acetonitrile, solvent C: 50 mM EDTA, 100 mM triethylamine, hydrochloric acid was used to adjust pH 7.2, 90% 18.2 MΩ MilliQ water/ 10% methanol, HPLC condition: 100% C for 8 min to remove excess Cu ion, then 90% A + 10% B for 3 min, and then 1% per min gradient B for 16 min) to afford desired products.

Product **1**, 22% GC HP (5'- cRGD/Cy3B, 3'- NH_2): MS (ESI) calcd. For $[M]^+$: 10006.7; deconvoluted: 10005.5

Product **2**, 100% GC HP (5'- cRGD/Cy3B, 3'- NH_2): MS (ESI) calcd. For $[M]^+$: 11004.4; deconvoluted: 11004.4

2. **NHS reaction for amine-modified oligonucleotides**

A solution of 3'-amine modified oligonucleotide in 18.2 MΩ MilliQ water (10 µL) was added 10 µL of 10X PBS and 10 µL of an aqueous solution of 1 M $NaHCO_3$. The mixture was

then added to sulfoNHS-DBCO or NHS-DBCO (Click Chemistry Tools, 300 μg) for 22% HP and 100% HP, respectively, in DMSO (70 μL) and left for 1.5 hours. The product was filtered through a microcentrifuge filter (0.22 μm) with 50 μL of 18.2 MΩ MilliQ water. The filtrate was subjected to reverse-phase HPLC (AdvancedBio Oligonucleotides, Agilent, 4.6 × 150 mm, 0.5 mL min^{-1} flow rate; solvent A: 0.1 M triethylammonium acetate (TEAA), solvent B: acetonitrile; starting condition: 82% A + 18% B, 0.83% per min gradient B) to desired products.

Product **3**, 22% GC HP (5'- cRGD/Cy3B, 3'- DBCO): MS (ESI) calcd. For $[M]^+$: 10320.1; deconvoluted: 10312.2

Product **4**, 100% GC HP (5'- cRGD/Cy3B, 3'- DBCO): MS (ESI) calcd. For $[M]^+$: 11291.8; deconvoluted: 11291.6

3. Strain-promoted azido-alkyne cyclization reaction for alkyne-modified oligonucleotides (22% GC hairpin & 100% GC hairpin)

To a solution of DBCO-modified oligonucleotides in 18.2 MΩ MilliQ water (20 μL) was added large excess amount of BHQ2/MeTz-N_3 or MeTz-PEG_4-N_3 (Click Chemistry Tools) in DMSO (80 μL) and left for 3 hours. The resulting solution was filtered through a microcentrifuge filter (0.22 μm). The filtrate was subjected to reverse-phase HPLC (AdvancedBio Oligonucleotides, Agilent, 4.6 × 150 mm, 0.5 mL min^{-1} flow rate; solvent A: 0.1 M triethylammonium acetate (TEAA), solvent B: acetonitrile; starting condition: 75% A + 25% B, 1% per min gradient B for 30 min) to PS modified tension probes.

Product **5**, 22% GC HP Probe (5'- cRGD/Cy3B, 3'- BHQ2/MeTz): MS (ESI) calcd. For $[M]^+$: 11328.2; deconvoluted: 11327.1

Product **6**, 100% GC HP (5'- cRGD/Cy3B, 3'- BHQ2/MeTz): MS (ESI) calcd. For $[M]^+$: 12297.9; deconvoluted: 12297.2

Product **7**, 22% GC HP Probe (5'- cRGD/Cy3B, 3'- MeTz): HPLC starting condition: 75% A + 25% B, 0.5% per min gradient B for 25 min: MS (ESI) calcd. For $[M]^+$: 10711.5; deconvoluted: 10710.8

4. Synthesis of oligonucleotides for control experiments(22% GC hairpin)

A solution of 3'-amine modified oligonucleotide in 18.2 MΩ MilliQ water (10 μL) was added 2.5 μL of 10X PBS, 2.5 μL of an aqueous solution of 1 M $NaHCO_3$, and 5 μL of 18.2 MΩ MilliQ water. The mixture was then added NHS-QSY9 (50 μg) or NHS-Cy3B (50 μg) for Product **1** or 22% HP, respectively, in DMSO (5 μL) and left for 1 hour. The product was filtered through a P2 gel with 75 μL of 18.2 MΩ MilliQ water. The filtrate was subjected to reverse-phase HPLC (AdvancedBio Oligonucleotides, Agilent, 4.6 × 150 mm, 0.5 mL min^{-1} flow rate; solvent A: 0.1 M triethylammonium acetate (TEAA), solvent B: acetonitrile) to desired products.

Product **8**, 22% GC HP Probe (5'- cRGD/Cy3B, 3'- QSY9): HPLC starting condition: 82.5% A + 17.5% B, 0.5% per min gradient B for 20 min: MS (ESI) calcd. For $[M]^{2+}$: 10807.6; deconvoluted: 10807.8

Product **9**, 22% GC HP Probe (5'- Alkyne, 3'- Cy3B): HPLC starting condition: 83% A + 17% B, 1% per min gradient B for 23 min: MS (ESI) calcd. For $[M]^+$: 8957.9; deconvoluted: 8958.7

MeTz-Lys(N_3)-BHQ2 (BHQ2/MeTz-N_3) was ligated to the corresponding 5' alkyne-modified strand (Product **9**) via 1,3-dipolar cycloaddition reaction. Briefly, a solution of Product **9** was reacted 1 hour at 50 ºC with 20 nmol of azido reagents in the presence of sodium ascorbate (0.10 µmol), $CuSO_4$ (0.10 µmol), THPTA (0.15 µmol), and triethyl amine (4 µmol) in 45 µL (1: 4 = 18.2 MΩ MilliQ water: DMSO). The product was filtered through a microcentrifuge filter (0.22 µm). The filtrate was subjected to reverse-phase HPLC (AdvancedBio Oligonucleotides, Agilent, 4.6 × 150 mm, 0.5 mL min^{-1} flow rate; solvent A: 0.1 M triethylammonium acetate (TEAA), solvent B: acetonitrile, solvent C: 50 mM EDTA, 100 mM triethylamine, hydrochloric acid was used to adjust pH 7.2, 90% 18.2 MΩ MilliQ water/ 10% methanol, HPLC condition: 100% C for 8 min to remove excess Cu ion, then 90% A + 10% B for 3 min, and then 2% per min gradient B for 16 min,) to afford desired products.

Product **10**, 22% GC HP Probe (5'- BHQ2/MeTz, 3'- Cy3B): HPLC condition: 100% C for 8 min to remove excess Cu ion, then 85% A + 15% B for 3.5 min, and then 2% per min gradient B for 25 min, MS (ESI) calcd. For $[M]^+$: 9964.0; deconvoluted: 9965.1

5. Thermal Melting Analysis

For the experimental thermal melting curve, 100 µL solutions of compound **8** at 20 µM were prepared in 1X PBS in qPCR tubes. The probe solutions were heated to 70 °C for 3 minutes and then cooled at a rate of 1.3 °C min^{-1} to 25 °C to hybridize. The solutions were then transferred to the 4 wells of a 96-well qPCR plate in 20 µL each for three

individual measurements for a condition. Using the qPCR instrument (Light Cycler 96), the plate was incubated at 37 ºC for 5 min and then heated to 95 ºC over 1900 seconds with Cy3B fluorescent measurements.

Because the thermodynamic equilibrium of DNA folded structure is an intramolecular transition, their thermodynamic parameters, including ΔG, ΔH, and ΔS, are concentration-independent.[1] Thus, the probe's van't Hoff equation for thermodynamic analysis can be adopted from the definition of Gibbs free energy equation.

6. Estimation of $F_{1/2}$ at which 50% of hairpins unfold at the force

$F_{1/2}$ of 22% GC content DNA HP was estimated based on the work of Woodside et. al. 2006 PNAS,[2] and derived from the worm-like chain model using the following equation.

$$F_{1/2} = \frac{\Delta G_{mfold} + \Delta G_{Stretch}}{\Delta x} \quad \Delta G_{Stretch} = \frac{k_B T}{L_p} \frac{L_0}{4(1-x/L_0)} [3(x/L_0)^2 - 2(x/L_0)^3]$$

where, ΔG_{mfold} is the delta free energy of hairpin folding calculated by thermodynamic measurement, $\Delta G_{Stretch}$ is the delta free energy of stretching the ssDNA, and Δx is the hairpin displacement for unfolding estimated by the equation of $0.44 \times (n-1) - 2$ (nm). n is the number of nucleotides, composing of DNA hairpin structure. L_p is the persistence length of ssDNA (~1.3 nm), L_0 is the contour length of ssDNA (0.63 nm per nucleotide), x is the hairpin extension from equilibrium and was calculated by using $(0.44 \times (n-1))$ nm, k_B is the Boltzmann constant, and T is temperature in kelvin.

7. Electron Spray Ionization (ESI) mass spectroscopy

The molecular weight of the products was evaluated with an electron spray ionization (ESI) method using a Thermo Fisher Scientific Orbitrap. For the small molecules, the samples were prepared in the 18.2 MΩ MilliQ water and recorded the spectra in positive charge mode eluted with a mixture of 60% of 18.2 MΩ MilliQ water and 40% of acetonitrile containing 0.05% formic acid. For oligonucleotides, the samples were prepared in the mixture of 70% 18.2 MΩ MilliQ water and 30% methanol containing 10 μM ethylenediaminetetraacetic acid (EDTA), 0.0375% triethylamine, and 0.75% of 1,1,1,3,3,3-hexafluoro-2-propanol (HFIP) and recorded the spectra with negative charge mode eluted with same solution.[3] The obtained ESI-MS spectrum (*m/z*) was then deconvoluted for the main peak to obtain average molecular weight for the oligonucleotides.

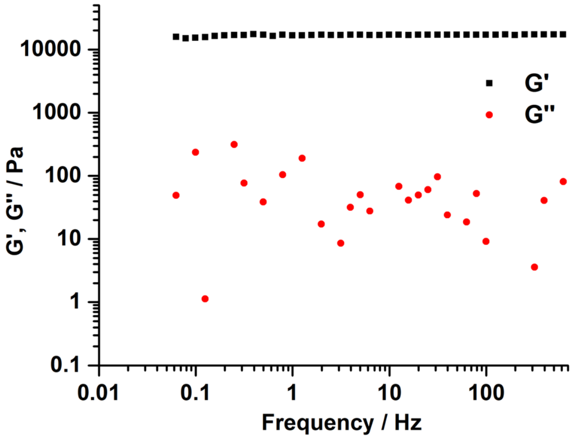

Figure S1. Frequency scan (0.1-1000 Hz) in rheology characterization of a PEG hydrogel that has 13 kPa modulus. Note that G’>G’’ in all frequencies, which represents a classic hydrogel behavior.

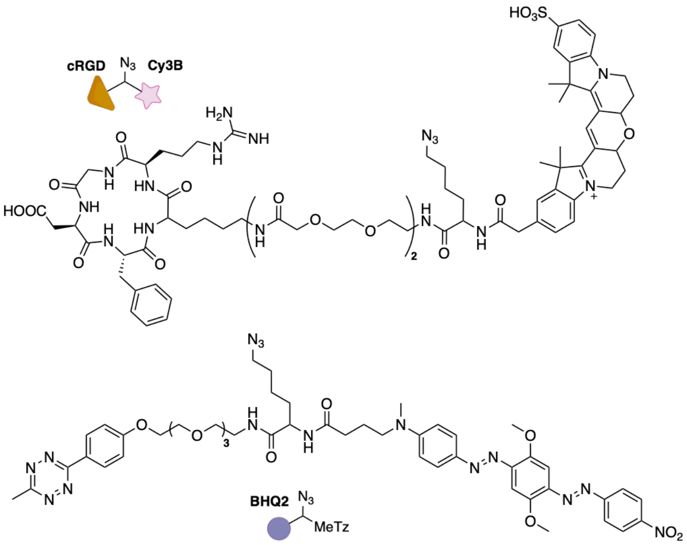

Figure S2. Chemical structures of **(top)** cRGD/Cy3B-N_3 and **(bottom)** BHQ2/MeTz-N_3 conjugates.

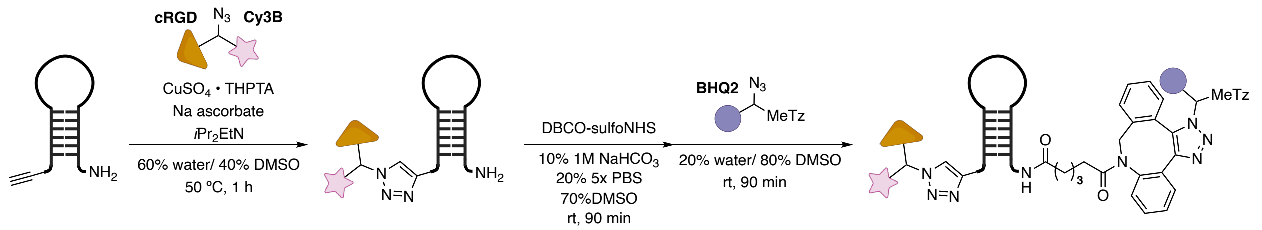

Scheme S1. Synthetic scheme of PS-modified DNA HP probes

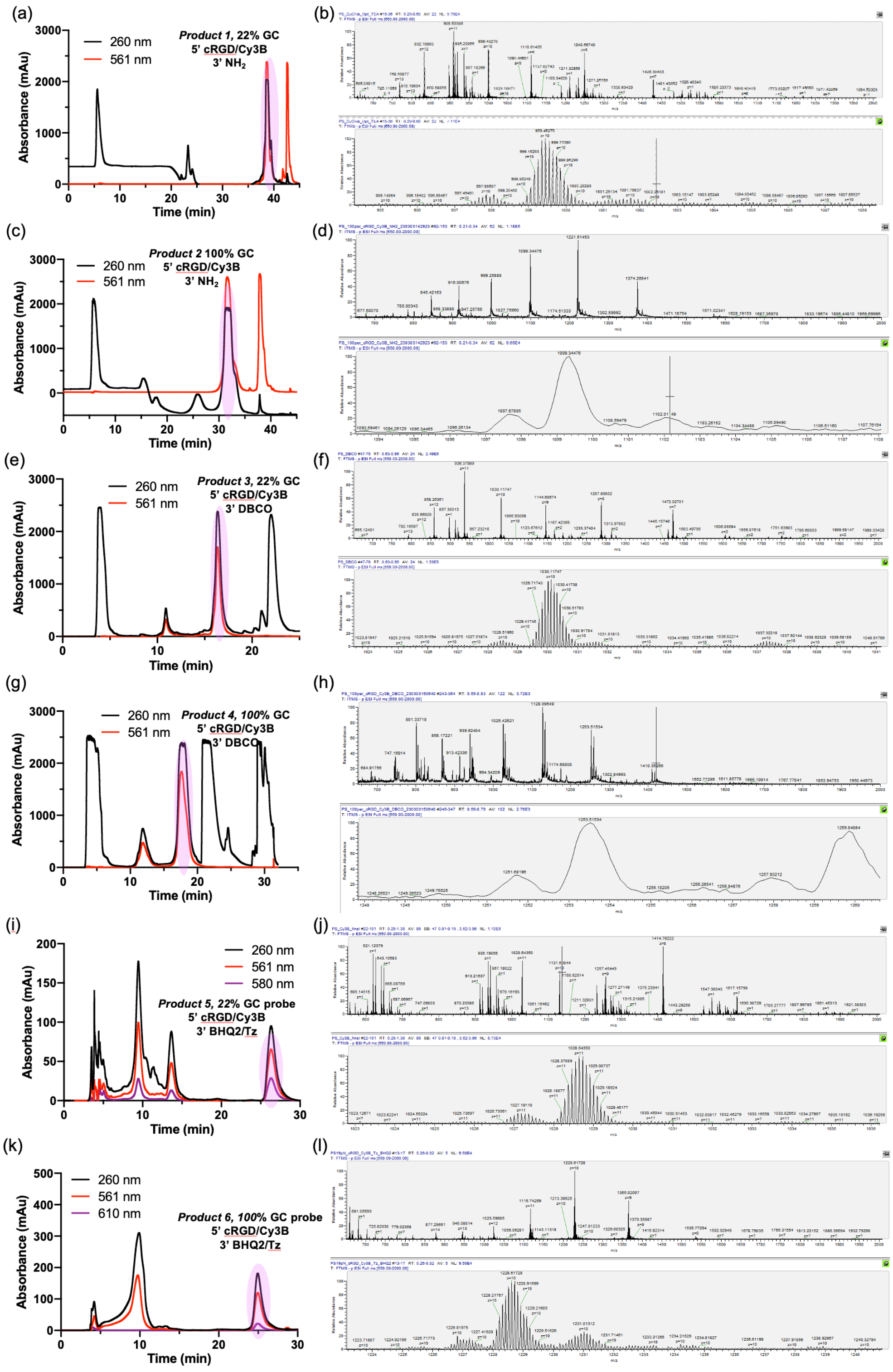
(a)
260 nm
561 nm
Product 1, 22% GC
5' cRGD/Cy3B
3' NH$_2$
Absorbance (mAu)
Time (min)
(b)
(c)
260 nm
561 nm
Product 2 100% GC
5' cRGD/Cy3B
3' NH$_2$
Absorbance (mAu)
Time (min)
(d)
(e)
260 nm
561 nm
Product 3, 22% GC
5' cRGD/Cy3B
3' DBCO
Absorbance (mAu)
Time (min)
(f)
(g)
260 nm
561 nm
Product 4, 100% GC
5' cRGD/Cy3B
3' DBCO
Absorbance (mAu)
Time (min)
(h)
(i)
260 nm
561 nm
580 nm
Product 5, 22% GC probe
5' cRGD/Cy3B
3' BHQ2/Tz
Absorbance (mAu)
Time (min)
(j)
(k)
260 nm
561 nm
610 nm
Product 6, 100% GC probe
5' cRGD/Cy3B
3' BHQ2/Tz
Absorbance (mAu)
Time (min)
(l)

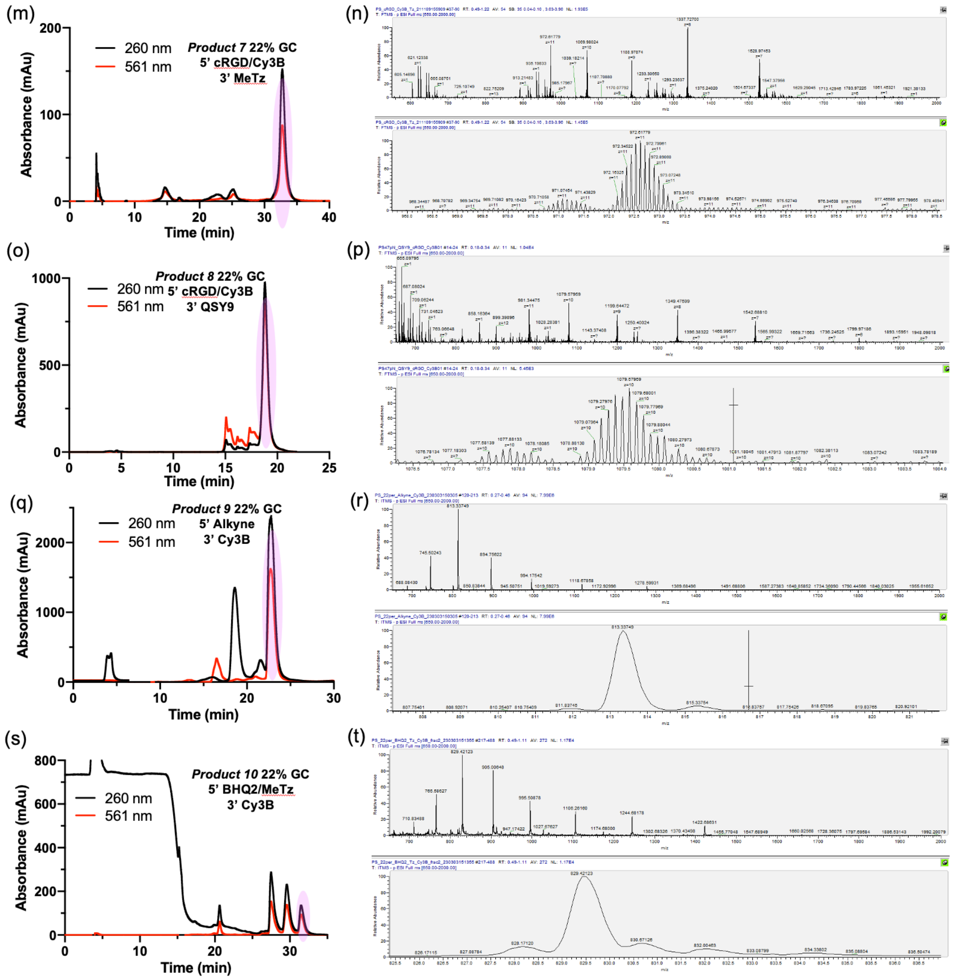

Figure S3. HPLC chromatogram and ESI-MS characterization of synthesized product. (a) HPLC chromatogram of product **1**, (b) ESI MS of product **1**, (c) HPLC chromatogram of product **2**, (d) ESI MS of product **2**, (e) HPLC chromatogram of product **3**, (f) ESI MS of product **3**, (g) HPLC chromatogram of product **4**, (h) ESI MS of product **4**, (i) HPLC chromatogram of product **5**, (j) ESI MS of product **5**, (k) HPLC chromatogram of product **6**, (l) ESI MS of product **6**, (m) HPLC chromatogram of product **7**, (n) ESI MS of product **7**, (o) HPLC chromatogram of product **8**, (p) ESI MS of product **8**, (q) HPLC chromatogram of product **9**, (r) ESI MS of product **9**, (s) HPLC chromatogram of product **10**, (t) ESI MS of product **10**.

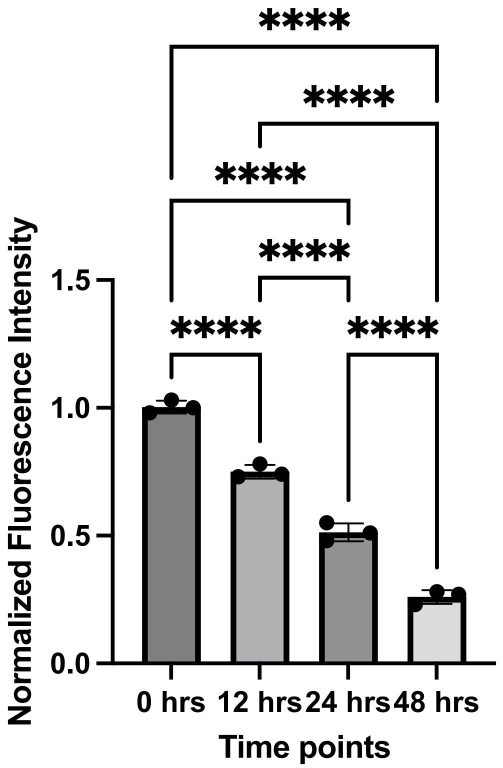

Figure S4. Loss of fluorescence signals for DNA probes tethered with Biotin-Streptavidin. Bar graphs show loss in fluorescence signal of hydrogels coated biotin and streptavidin attachment. ****, ***, **, * and ns indicate p<0.0001, p<0.001, p<0.01, p<0.05, and not significant, respectively, as determined from one-way ANOVA. Error bars show the standard deviation for *N*=3.

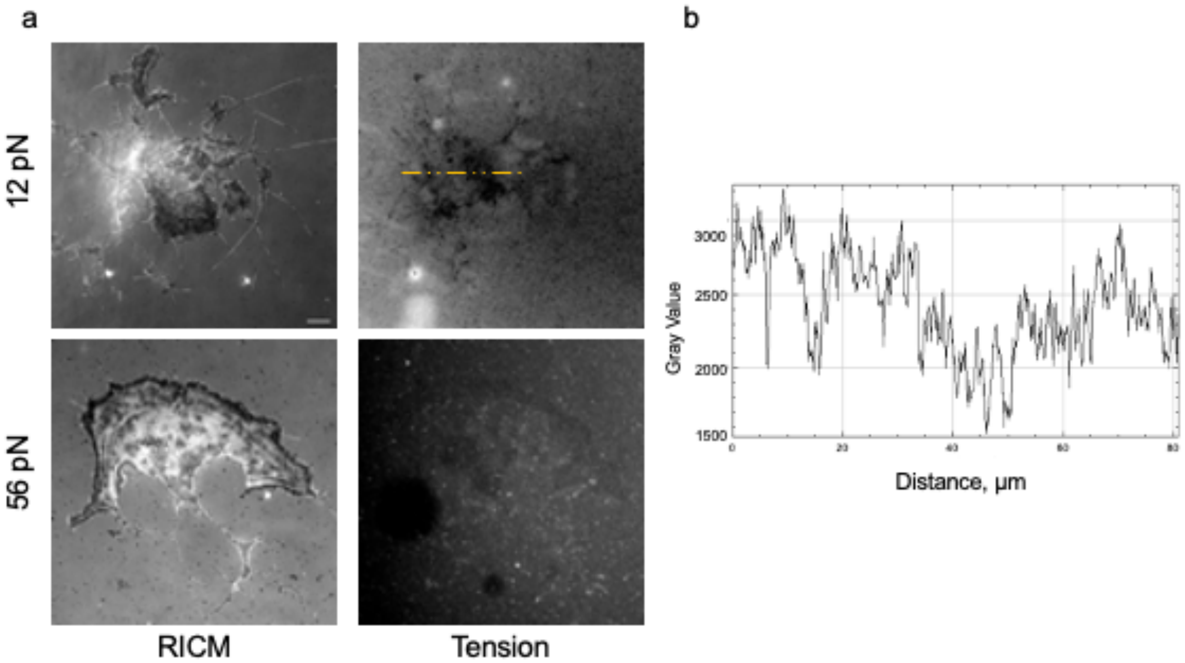

Figure S5. Conventional DNA duplex probes (TGT) showed HeLa cell-induced probe degradation on the glass surface. a. Representative RICM and fluorescent tension images of HeLa cells cultured on glass surface coated with TGT. Images were taken 3 hrs after cell seeding on biotin-tethered 12 and 56 pN TGT probes. b. Line scan of fluorescent tension image. Loss of fluorescent signal intensity indicates HeLa cells degrade the TGT probes due to nuclease and protease activities. Scale bar- 10 µm.

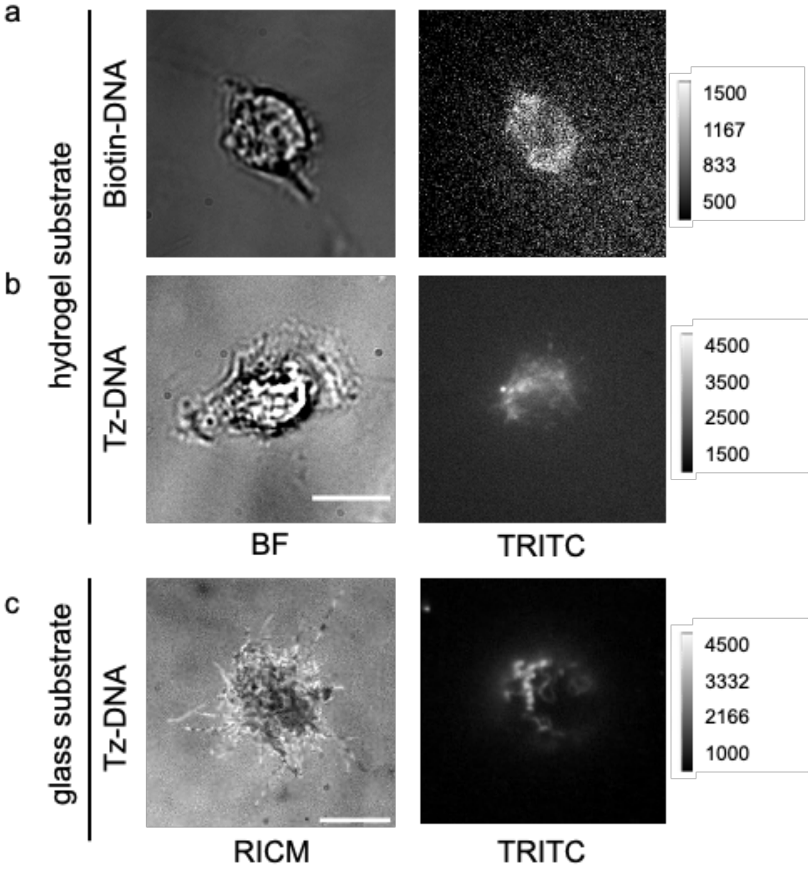

Figure S6. HeLa cells internalized the fluorescent tension probes when cells are cultured on the conventional DNA HP probe-tethered hydrogel surface or glass substrate. **a**, **b**. Representative BF and TRITC images of HeLa cells cultured on conventional DNA probes tethered with Biotin-streptavidin interaction or anchored covalently using TCO-Tz coupling. **c**. Representative RICM and TRITC images of HeLa cells cultured on conventional DNA probes anchored covalently to the glass substrate using TCO-Tz coupling. Scale bar- 10 μm.

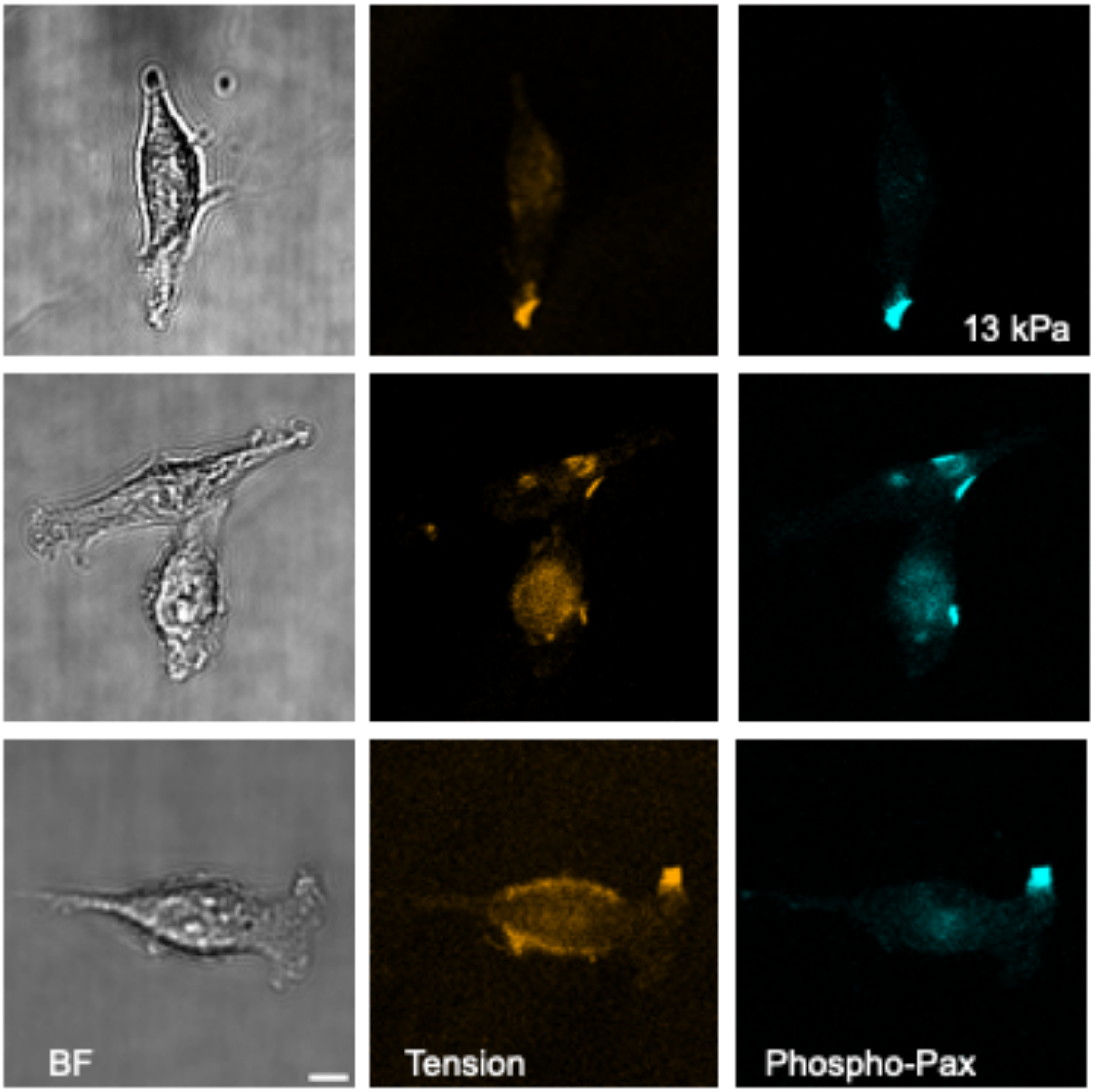

Figure S7. P-Tyr118 paxillin and fluorescent tension signal (PS DNA HP probe, 22% GC content) showed good colocalization. Three representative images of Immunostained HeLa cells cultured on 13 kPa hydrogels coated with PS DNA HP probes show tension signal is colocalized with phospho-paxillin signals, suggesting the focal adhesion formation under tension signal. Scale Bar- 10 μm.

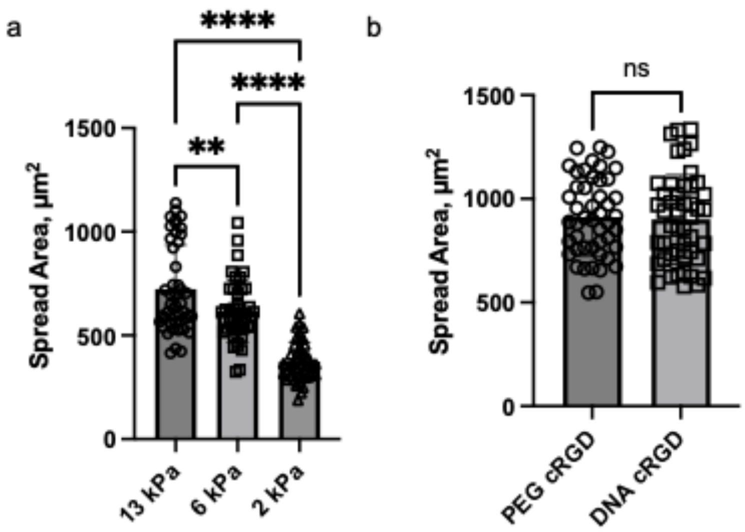

Figure S8. Spread area of HeLa cells cultured on different types of hydrogel surfaces. a. Bar graph plotting spread area of HeLa cells on 13, 6, and 2 kPa hydrogels coated with PS DNA HP probe (22% GC content). b. Bar graph showing HeLa cells show comparable spreading area when cultured on the hydrogel coated with PS DNA HP probe or coated with cRGD and cRGD-lacked PS DNA HP. ****, ***, **, * and ns indicate $p<0.0001$, $p<0.001$, $p<0.01$, $p<0.05$, and not significant, respectively, as determined from one-way ANOVA. Error bars show the standard deviation for *N*=3.

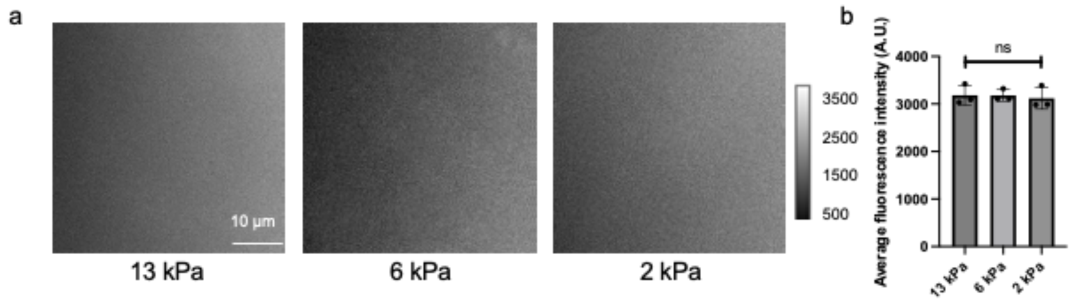

Figure S9. DNA density confirmation on 13, 6, and 2 kPa hydrogels. a. Representative TRITC images of 13, 6, and 2 kPa hydrogels. b. Bar graph showing average fluorescent intensity of PS DNA HP probe tethered hydrogels. The data with identical background intensity indicates similar DNA density for all hydrogels used in this study. ns indicate not significant with $p>0.5$, as determined from one-way ANOVA. Error bars show the standard deviation for *N*=3.

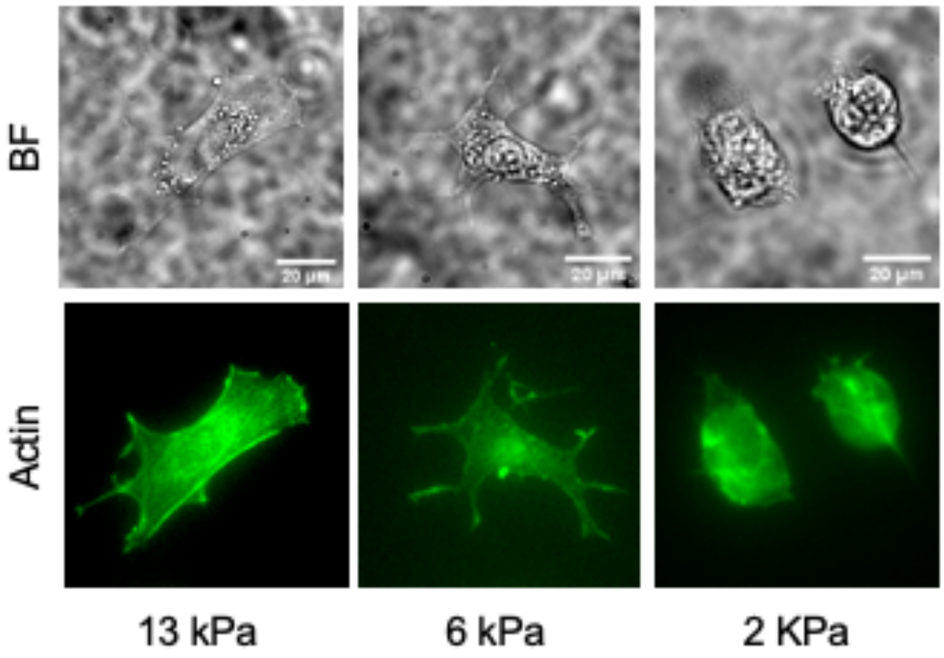

Figure S10. F-actin expression of HeLa cells. a. Representative BF and Actin images of HeLa cells cultured on 13, 6 and, 2 kPa hydrogels with PS DNA HP probe (22% GC content). F-actin was stained with Phalloidin-Alexa Fluor 488. Scale bar- 20 µm

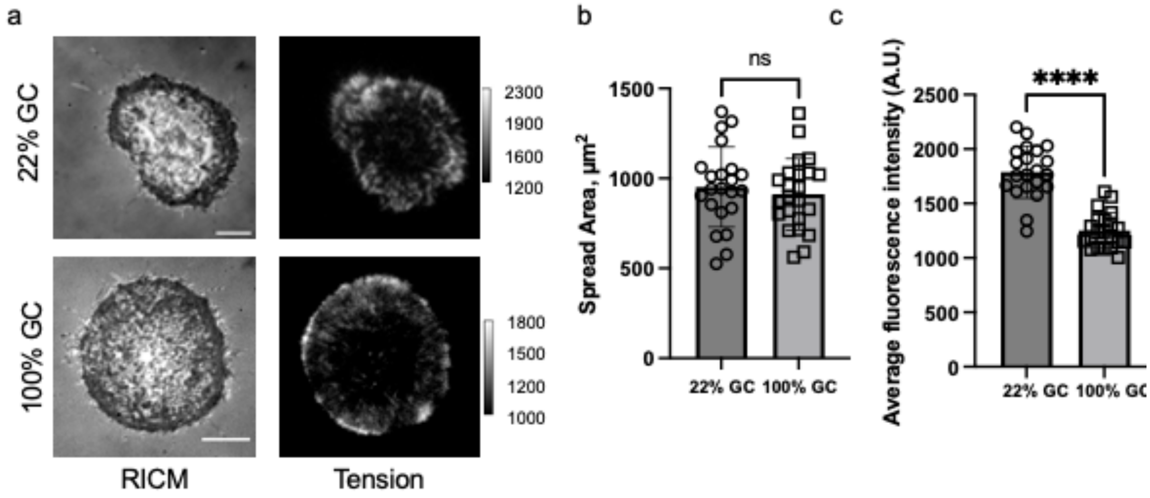

Figure S11. HeLa cells cultured on PS DNA HP probes (22% and 100% GC content) tethered to glass substrate showed fluorescent tension signal. a. Representative RICM and fluorescent tension signals of heLa cells cultured on 22% and 100% GC content probes. b, c. Bar graphs plotting spread area and average tension signals of HeLa cells on two different HP probes show cells have similar spread area, but HeLa cells have greater tension on 5.8 pN probes.****, and ns indicate p<0.0001, and not significant respectively, as determined from one-way ANOVA. Scale Bar- 10 μm

References

1. Mergny, J. L.; Lacroix, L., Analysis of thermal melting curves. *Oligonucleotides* **2003,** *13* (6), 515-537.

2. Woodside, M. T.; Behnke-Parks, W. M.; Larizadeh, K.; Travers, K.; Herschlag, D.; Block, S. M., Nanomechanical measurements of the sequence-dependent folding landscapes of single nucleic acid hairpins. *Proc. Natl. Acad. Sci. U S A* **2006,** *103* (16), 6190-6195.

3. Hail, M. E.; Elliott, B.; Anderson, K., High-Throughput Analysis of Oligonucleotides Using Automated Electrospray Ionization Mass Spectrometry. *Am. Biotechnol. Lab.* **2004,** *12* (12), 12–14.
